# Impact of Cytogenetic Response to Therapy on Long‐Term Survival in Acute Myeloid Leukemia

**DOI:** 10.1002/ajh.70000

**Published:** 2025-07-11

**Authors:** John Hanna, Emily C. Zabor, Moath Albliwi, Jessica El‐Asmar, Daniel P. Nurse, Ameed Bawwab, Hasan Abuamsha, Yomna Abu‐Farsakh, Heya Batah, Asad Rauf, Joy Nakitandwe, David S. Bosler, Akriti G. Jain, John C. Molina, Sophia Balderman, Abhay Singh, Aaron T. Gerds, Sudipto Mukherjee, Ronald M. Sobecks, Anjali S. Advani, Hetty E. Carraway, Caroline Astbury, Moaath K. Mustafa Ali

**Affiliations:** ^1^ Department of Internal Medicine Cleveland Clinic Cleveland Ohio USA; ^2^ Department of Quantitative Health Sciences Cleveland Clinic Taussig Cancer Institute Cleveland Ohio USA; ^3^ Department of Hematology and Medical Oncology Cleveland Clinic Taussig Cancer Institute Cleveland Ohio USA; ^4^ Department of Pathology and Laboratory Medicine Cleveland Clinic Diagnostics Institute Cleveland Ohio USA; ^5^ Department of Hematology and Medical Oncology Cleveland Clinic Taussig Cancer Institute, Blood and Marrow Transplant Program Cleveland Ohio USA

**Keywords:** acute myeloid leukemia, cytogenetic response, karyotype, prognosis, survival

## Abstract

Prognostication in acute myeloid leukemia (AML) relies on clinical, molecular, and cytogenetic factors. In this retrospective study, we examined the impact of different levels of cytogenetic response on overall survival (OS) and event‐free survival (EFS) in AML. Among 973 adult AML patients treated at Cleveland Clinic (5/2017–9/2023), 563 patients had baseline cytogenetic data and post‐treatment response assessment available. Based on baseline and response cytogenetic status, patients were categorized into: normal to normal (NL‐Cy to NL‐Cy, *n* = 221, 39%), normal or abnormal to gain (NL/Abnl‐Cy to Gain‐Cy, *n* = 46, 8.2%), abnormal to persistent (Abnl‐Cy to Persistent‐Cy, *n* = 81, 14%), abnormal to partial response (Abnl‐Cy to Partial‐Cy, *n* = 20, 3.6%), and abnormal to complete response (Abnl‐Cy to NL‐Cy, *n* = 195, 35%). Landmark analysis was used to account for post‐treatment assessments. The cohort had a median age of 62 years (interquartile range: 52–69), 256 females (45%), 90% were White, and median follow‐up of 45.8 months (range: 0.73–191.3). The median OS and hazard ratios (HRs) from multivariable regression analysis were as follows: NL‐Cy to NL‐Cy: 37 months (95% CI: 27–91), HR = reference; NL/Abnl‐Cy to Gain‐Cy: 14 months (95% CI: 8.6–30), HR = 1.5 (95% CI: 0.99–2.39); Abnl‐Cy to Persistent‐Cy: 13 months (95% CI: 12–18), HR = 1.61 (95% CI: 1.13–2.31); Abnl‐Cy to Partial‐Cy: 25 months (95% CI: 14‐NC), HR = 0.76 (95% CI: 0.39–1.49); and Abnl‐Cy to NL‐Cy: 27 months (95% CI: 19–101), HR = 1.25 (95% CI: 0.93–1.68) (*p* = 0.038). Achieving cytogenetic remission, complete or partial, was associated with better survival outcomes. These findings highlight the importance of monitoring cytogenetic responses to inform treatment decisions and support integrating cytogenetic response into risk‐adapted, personalized AML management strategies.

## 1 Introduction

Prognostication in acute myeloid leukemia (AML) depends on patient and disease‐specific factors such as age, performance status, molecular markers, and cytogenetic abnormalities. Among these, cytogenetic abnormalities remain one of the strongest predictors of prognosis [1]. Cytogenetic abnormalities are categorized into favorable, intermediate, and poor risk groups based on their prognostic significance in the most recent 2022 European Leukemia Network (ELN) risk classification [2]. Other baseline characteristics that have prognostic significance are the presence of pathogenic variants in several genes, including *NPM1*, *FLT3*, *CEBPA*, *TP53*, *ASXL1*, *EZH2*, *RUNX1*, *SF3B1*, *SRSF2*, *STAG2*, *U2AF1*, and *ZRSR2*. While identifying cytogenetic abnormalities at baseline has been recognized as a critical prognostic factor, increasing attention has shifted toward the dynamic assessment of cytogenetic response, specifically, the achievement of cytogenetic response following first‐line therapy.

Achieving a cytogenetic response after first‐line therapy, defined as the normalization or improvement of abnormal karyotypes, may reflect treatment efficacy and provide additional prognostic insight beyond molecular and clinical responses. Conversely, persistent cytogenetic abnormalities or the acquisition of new abnormalities, independent of blast and blood count changes, may indicate residual disease or heightened relapse risk, significantly influencing survival outcomes. Early work by cooperative research groups investigated the importance of assessing cytogenetic status at the time of morphologic complete remission (CR). In these studies, persistence of cytogenetic abnormalities at CR was consistently linked to higher rates of relapse compared to patients who achieved complete cytogenetic response [3, 4, 5]. The prognostic impact of cytogenetic response is further highlighted in the context of allogeneic hematopoietic cell transplantation (HCT). Multiple studies indicate that residual cytogenetic disease present before HCT predicts higher post‐transplant relapse rates and decreased survival, underscoring the necessity of achieving complete cytogenetic response before transplantation whenever possible [4, 6, 7].

Many prior studies evaluating the prognostic value of cytogenetic response in adult AML have focused primarily on the presence or absence of residual cytogenetic abnormalities at remission, often dichotomizing patients by whether their post‐treatment karyotype remains abnormal or reverts to normal [3, 4, 5]. However, these approaches frequently do not distinguish between the persistence of diagnostic clones and the emergence of new, pathogenic cytogenetic abnormalities during treatment. The clinical significance of clonal evolution, specifically, the gain of novel cytogenetic lesions after induction, has increasingly been recognized as a marker of adverse prognosis, yet remains underexplored in most survival models. Previous research examined the clinical significance of clonal evolution, defined as new somatic mutations, but the significance of cytogenetic evolution at the time of response assessment is less clear [8, 9]. In a study by Murray et al. [10], they showed that cytogenetic evolution is a common feature at the time of relapse or refractory disease. An analytic approach that explicitly categorizes both the persistence of original cytogenetic abnormalities and the acquisition of new clones post‐therapy would provide a more comprehensive assessment of clonal dynamics. Another limitation of existing literature on cytogenetic remission is that the clinical significance of partial cytogenetic abnormality resolution remains undetermined. Moreover, it is unknown if the assessment of cytogenetic response retains its prognostic significance when accounting for other baseline variables, including comorbidities and relevant mutations that were introduced in the prognostic classification in 2022.

While achieving complete cytogenetic remission has been associated with improved outcomes and lower relapse risk, further research is necessary to determine the prognostic implications of other levels of response on long‐term survival in AML patients. This study aims to examine the impact of different levels of cytogenetic response after first‐line treatment on overall survival (OS), event‐free survival (EFS), and clinical response rates in patients diagnosed with AML. We categorized and analyzed different response levels to address gaps in prior research, which have often overlooked the distinct outcomes associated with the development of new cytogenetic abnormalities during treatment and the partial resolution of abnormalities. By identifying the prognostic role of cytogenetic response, this work aims to refine risk stratification and improve therapeutic decision‐making in adult AML management.

## 2 Methods

### 2.1 Study Design, Setting, and Participants

This is a retrospective cohort study conducted at all the Cleveland Clinic centers in Ohio, examining the impact of cytogenetic response on long‐term survival in adult patients with AML. The study included all patients diagnosed with AML from May 2017 to September 2023. Only patients with available cytogenetic exams at baseline and response assessment were analyzed. Data was last updated in January 2025. Ethical approval was obtained from the Cleveland Clinic Institutional Review Board.

### 2.2 Data Source and Measurements

Patient data were retrieved from the electronic healthcare records and the tumor registry. Each patient's data was extracted by a medical doctor from Epic electronic medical records. To minimize missing data, we reviewed Care Everywhere, Epic's data‐sharing platform. Data quality and accuracy were checked using various tools, including Excel filters, and with assistance from the biostatistician. At least one additional chart review was conducted to address any missing data. Relevant demographic and clinical characteristics, including age, sex, ethnicity, comorbidities, AML subtypes, antecedent myelodysplastic syndrome/myeloproliferative neoplasm (MDS/MPN), bone marrow biopsy and aspirate findings, baseline cytogenetic exam, *FLT3* mutational status by fragment segment polymerase chain reaction (PCR), molecular profile by next‐generation sequencing (NGS), baseline laboratory results, all therapeutic lines, response outcomes, and survival outcomes, were recorded. Comorbidities included: coronary artery disease, congestive heart failure, diabetes mellitus, hypertension, hyperlipidemia, lung disease, chronic kidney disease, cirrhosis, cerebrovascular disease, and rheumatologic disorders. The AML subtypes were determined using revised WHO 2016, WHO 2022, and the 2022 International Consensus Classification of Myeloid Neoplasms [11, 12, 13]. Extracted data was stored in REDcap, a secure web application for managing databases [14].

### 2.3 Outcomes and Response Assessment

The outcomes of our study included composite response rate (CCR), OS, and EFS. Treatment responses were assessed according to the 2022 ELN criteria: CCR, which included complete response (CR) and complete response with incomplete hematologic recovery (CRi) [15]. Response landmark was determined per ELN 2022 guidelines [12]. For intensive chemotherapy, the assessment was conducted after one or two cycles. For venetoclax plus hypomethylating agents (Ven + HMA), the response was assessed after 3 months, and for single‐agent hypomethylating therapy, the response evaluation occurred at 6 months. Other treatment regimens followed the time points established in the respective first‐line registration trials.

### 2.4 Cytogenetic Exam and Cytogenetic Response Comparison Groups

The cytogenetic exams were performed using bone marrow aspirates or peripheral blood when bone marrow aspirates were unavailable. All cytogenetic exams were reviewed by experienced cytogeneticists at the time of diagnosis and response assessment. Fluorescence in situ hybridization (FISH) results were included when available. The AML FISH panel used at Cleveland Clinic included probes targeting common recurrent rearrangements, including PML::RARA [t(15;17)], KMT2A (11q) breakapart, CBFB (16q) breakapart, and RUNX1::RUNX1T1 [t(8;21)]. Not all patients had a FISH test, and its use was based on the discretion of the treating leukemia specialist and when recommended by pathology.

An adequate cytogenetic exam was defined as one where at least 20 metaphase cells were analyzed. The categorization of baseline cytogenetic response was: (1) not performed, (2) poor banding, inadequate, (3) normal, inadequate (< 20 metaphases counted from bone marrow sample), (4) normal, suboptimal (≥ 20 metaphases counted from peripheral blood sample), (5) normal karyotype (no cytogenetic abnormality, ≥ 20 metaphases counted from bone marrow sample), (6) abnormal (≥ 20 or < 20 metaphases), and (7) abnormal FISH finding but normal karyotype (≥ 20 metaphases). In this study, categories 3, 4, and 5 were considered normal exams, and categories 6 and 7 were considered abnormal exams. The abnormal cytogenetic risk groups were classified into favorable, intermediate, and adverse using the 2022 ELN risk classification [2].

Constitutional (non‐pathogenic) findings were identified by board‐certified cytogeneticists based on their absence of known pathogenicity in AML and expert interpretation using established classification frameworks such as those from the Medical Research Council and European LeukemiaNet [2, 16]. Constitutional cytogenetic abnormalities were suspected if the finding was not a rearrangement known to be associated with neoplasia and was present in every cell. When suspected, cytogeneticists recommended confirmatory testing by performing chromosome analysis on stimulated peripheral blood.

No previously established cytogenetic response criteria had been defined or published before this study. For the purpose of this study, the categorization of cytogenetic response was standardized following discussions among leukemia specialists, molecular pathologists, and cytogeneticists. Table S1 summarizes the reasoning for the proposed categories. The criteria developed are as follows:

- **Normal Cytogenetic Exam (NL‐Cy)**: No pathogenic abnormalities were detected at the landmark evaluation. In cases where the exam was suboptimal (i.e., fewer than 20 metaphases analyzed) or based on peripheral blood, it was still classified as NL‐Cy for the purpose of this study.
- **Abnormal Cytogenetic Exam (Abnl‐Cy)**: Pathogenic abnormalities were identified by the cytogeneticist. Non‐pathogenic findings were excluded (e.g., inv(9)(p12q13)). This category primarily applied to patients with a normal baseline cytogenetic exam who later developed detectable abnormalities.
- **Persistent Cytogenetic Abnormalities (Persistent‐Cy)**: No reduction in abnormal clones and no emergence of normal clones. Defined as ≥ 50% abnormal metaphases.
- **Partial Cytogenetic Remission (Partial‐Cy)**: A reduction in abnormal clones accompanied by a ≥ 50% increase in normal clones. Calculated as: normal clones/(normal + abnormal clones) ≥ 50%.
- **Gain of Cytogenetic Abnormality (Gain‐Cy)**: The emergence of new pathogenic abnormalities not present at baseline. Defined as ≥ 2 metaphases for gains, rearrangements, or partial losses (e.g., deletions), and ≥ 3 metaphases for whole chromosome losses (e.g., −5, −7). Typically applied to patients with an abnormal karyotype at baseline.

Cytogenetic response was assessed at a 60‐day landmark after the initiation of frontline treatment. Using baseline cytogenetic exam and response cytogenetic exam, we categorized the cytogenetic response comparison groups as follows:

- **NL‐Cy → NL‐Cy**: Baseline normal cytogenetics remain normal at landmark evaluation.
- **NL‐Cy → Abnl‐Cy**: Baseline normal cytogenetics develop new pathogenic abnormalities.
- **Abnl‐Cy → NL‐Cy**: Abnormal cytogenetics at baseline normalize.
- **Abnl‐Cy → Persistent‐Cy**: Baseline abnormalities persist with no reduction and no new normal clones.
- **Abnl‐Cy → Partial‐Cy**: Baseline abnormalities reduce with the appearance of new normal clones.
- **Abnl‐Cy → Gain‐Cy**: Baseline abnormalities persist with the acquisition of new pathogenic abnormalities

**FIGURE 1:**
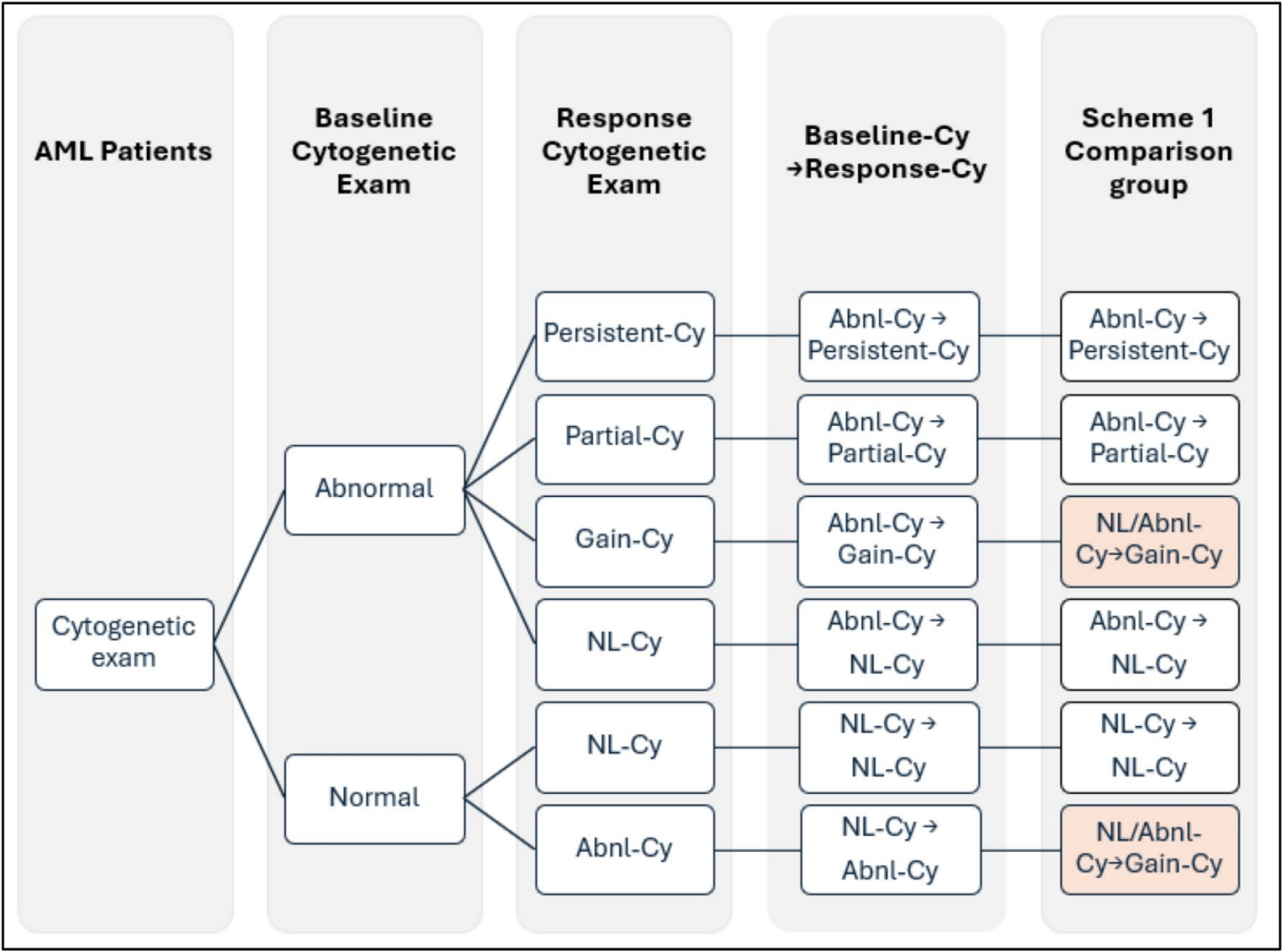
Comparison of patient groups with acute myeloid leukemia stratified by baseline and response cytogenetic state (analytic Scheme 1). Cy, cytogenetic; NL, normal; Abnl, abnormal. [Color figure can be viewed at wileyonlinelibrary.com]

In this study, we provided outcomes using two analytic schemes. Scheme one: We combined groups B and F and named the group NL/Abnl‐Cy → Gain‐Cy (Figure 1). Scheme two: We combined B, D, and F and named the group NL/Abnl‐Cy → Persistent/Gain‐Cy (Figure S1).

## 3 Statistical Analysis

Characteristics were summarized using median and interquartile ranges (IQR) and numbers with percentages, with univariable tests for differences using the Kruskal–Wallis test and Wilcoxon rank‐sum tests for continuous and categorical variables, respectively. OS time was calculated from the date of diagnosis to the date of death or last follow‐up, whichever came first. EFS was calculated from the date of diagnosis to the date of first refractory disease or relapse or death from any cause, and patients alive without refractory disease or relapse were censored at the last date their disease status was known. The Kaplan–Meier method estimated survival probabilities, and the log‐rank test was used to test for differences. Multivariable Cox proportional hazards (CPH) regression was used for multivariable analysis. We used a landmark analysis approach to account for the fact that cytogenetic response is assessed after treatment start. We used a landmark at 60 days after treatment initiation. Multivariable logistic regression (LR) analyzed associations with response. All adjustment variables, including genes, were selected a priori and included based on degrees of freedom for each endpoint. Tests for interaction between cytogenetic response groups and treatment type (intensive, Ven + HMA, Others) were based on models that included all adjustment variables except genetic factors due to sample size limitations. All statistical analyses were conducted using R version 4.4.0 [17].

## 4 Results

### 4.1 Study Population and Baseline Characteristics

A total of 973 patients with AML were treated at the Cleveland Clinic Ohio centers between 1/2015 and 9/2023. We excluded the following: 24 patients who lacked first‐line treatment details, 209 patients who had missing response cytogenetic exam, 155 who had response cytogenetic exam not performed, 14 patients who had poor banding, suboptimal response cytogenetic exam, and 6 patients who had missing baseline cytogenetic exam. The final analyzed sample included patients who had baseline and response cytogenetic exam available and consisted of 563 patients.

The median age at diagnosis was 62 years (IQR: 52–69), with a slight predominance of male patients (55%). The baseline demographic, clinical characteristics, and molecular features of the study cohort are summarized in Table 1. Baseline cytogenetic findings indicated that 41% of patients had a normal karyotype and 59% had abnormal cytogenetics at diagnosis. The median follow‐up time among survivors, from landmark, was 45.77 months (range: 0.73–191.31).

**TABLE 1:** Baseline demographic and clinical characteristics of acute myeloid leukemia patients at diagnosis included in the study.

Characteristic	*N* = 563^1^
Age at first diagnosis (years)	62 (52, 69)
Gender	
Female	256 (45%)
Male	307 (55%)
Race	
White	504 (90%)
Black	43 (7.7%)
Other	15 (2.7%)
Unknown	1
Coronary artery disease or cerebrovascular disease	65 (12%)
Chronic heart failure	34 (6.0%)
Diabetes mellitus	92 (16%)
Hypertension	247 (44%)
CKD stage 3/4/5/ESRD	31 (5.5%)
ECOG performance status	
0/1/2	551 (98%)
3/4	9 (1.6%)
Unknown	3
Blast percentage of peripheral blood	25 (8, 56)
Unknown	77
Blast percentage of bone marrow	48 (27, 72)
Unknown	23
Hemoglobin (g/dL)	8.40 (7.50, 9.80)
Unknown	12
WBC (× 10^3^/μL)	5 (2, 27)
Unknown	12
ANC (× 10^3^/μL)	1.0 (0.3, 3.2)
Unknown	39
Platelets (× 10^6^/μL)	56 (28, 102)
Unknown	13
Bone marrow cellularity	80 (50, 90)
Unknown	86
Antecedent MDS/MPN	
De novo AML	519 (92%)
MDS followed by AML	24 (4.3%)
MPN followed by AML	17 (3.0%)
MDS/MPN followed by AML	3 (0.5%)
Cytogenetics at baseline	
Abnormal (> or = 20 or < 20 metaphase)	321 (57%)
Abnormal FISH finding but Normal Karyotype (> or = 20 metaphases)	11 (2.0%)
Normal Karyotype (no cytogenetic abnormality, > or = 20 metaphases BM)	224 (40%)
Normal, Inadequate (< 20 metaphase BM)	5 (0.9%)
Normal, Suboptimal (> or = 20 metaphase PB)	2 (0.4%)
Cytogenetics at baseline, dichotomous	
Normal	231 (41%)
Abnormal	332 (59%)
Baseline *ASXL1*	
WT	327 (58%)
Mut	58 (10%)
Not tested	178 (32%)
Baseline *BCOR*	
WT	346 (61%)
Mut	36 (6.4%)
Not tested	181 (32%)
Baseline *CEBPA*	
WT	238 (42%)
Mut	23 (4.1%)
Not tested	302 (54%)
Baseline *EZH2*	
WT	365 (65%)
Mut	17 (3.0%)
Not tested	181 (32%)
Baseline *RUNX1*	
WT	320 (57%)
Mut	71 (13%)
Not tested	172 (31%)
Baseline *SF3B1*	
WT	358 (64%)
Mut	24 (4.3%)
Not tested	181 (32%)
Baseline *SRSF2*	
WT	327 (58%)
Mut	61 (11%)
Not tested	175 (31%)
Baseline *STAG2*	
WT	231 (41%)
Mut	29 (5.2%)
Not tested	303 (54%)
Baseline *TP53*	
WT	330 (59%)
Mut	57 (10%)
Not tested	176 (31%)
Baseline *U2AF1*	
WT	359 (64%)
Mut	25 (4.4%)
Not tested	179 (32%)
Baseline *ZRSR2*	
WT	370 (66%)
Mut	10 (1.8%)
Not tested	183 (33%)
Baseline *FLT3‐ITD* (PCR or NGS)	
Negative	370 (66%)
Positive	78 (14%)
Not tested	115 (20%)
First‐line treatment	
Intensive chemotherapy regimen	357 (63.4%)
Venetoclax + hypomethylating agent	73 (13%)
Others	133 (23.6%)

Abbreviations: ANC, absolute neutrophil count; BM, bone marrow; CKD, chronic kidney disease; ECOG, Eastern Cooperative Oncology Group; ESRD, end‐stage renal disease; FISH, fluorescence in situ hybridization; MDS, myelodysplastic syndrome; MPN, myeloproliferative neoplasm; Mut, mutation; NGS, next‐generation sequencing; PB, peripheral blood; PCR, polymerase chain reaction; WBC, white blood cells; WT, wild type.

^1^ Median (Q1, Q3); *n* (%).

Figure 1 illustrates the comparison groups in this analytic approach. At the 60‐day landmark, patients were categorized based on their cytogenetic response (Table S2). Of the 563 patients, 39% (*n* = 221) maintained normal cytogenetics (NL‐Cy to NL‐Cy), 8.2% (*n* = 46) had persistent or gained new abnormalities (NL/Abnl‐Cy to Gain‐Cy), 14% (*n* = 81) had persistent cytogenetic abnormalities (Abnl‐Cy to Persistent‐Cy), 3.6% (*n* = 20) showed partial remission (Abnl‐Cy to Partial‐Cy), and 35% (*n* = 195) showed complete cytogenetic response (Abnl‐Cy to NL‐Cy).

To test whether outcomes vary between first‐line therapy, we tested for interaction between the first‐line therapy and cytogenetic groups. The interaction was not significant (CCR interaction *p*‐value = 0.371; OS interaction *p*‐value = 0.579; EFS interaction *p*‐value = 0.631), indicating that the effect of cytogenetic response on outcome did not differ according to the treatment type received. Therefore, the rest of the results focus on the main effects models.

Table S3 summarizes a multivariable LR model for composite response rate for first‐line therapy in patients with AML, stratified by comparison groups in Scheme 1. The NL‐Cy to NL‐Cy category served as the reference group in this model. The NL/Abnl‐Cy to Gain‐Cy group had significantly lower odds of achieving response (odds ratio (OR): 0.09, 95% CI: 0.04–0.22). Similarly, patients who had Abnl‐Cy to Persistent‐Cy also demonstrated a significantly decreased odds of AML response (OR: 0.14, 95% CI: 0.07–0.28). On the other hand, the Abnl‐Cy to Partial‐Cy group had similar odds of response rate to the reference group (OR: 1.21, 95% CI: 0.38–4.45). Lastly, the Abnl‐Cy to NL‐Cy group odds of responding were not different from the reference group (OR: 1.50, 95% CI: 0.86–2.66).

**FIGURE 2:**
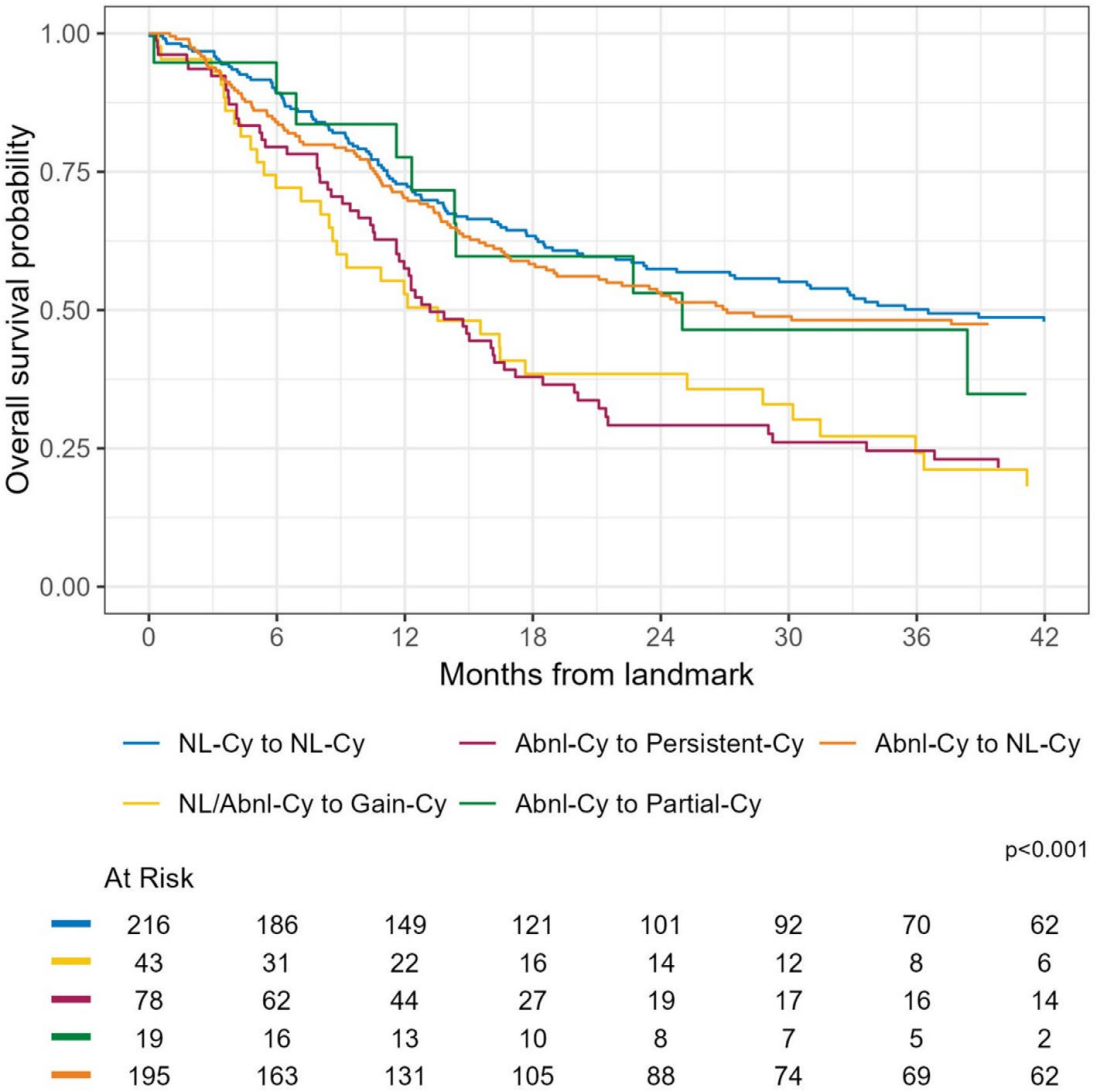
Kaplan–Meier survival curve for overall survival in acute myeloid leukemia patients stratified by baseline and response cytogenetic state (analytic Scheme 1). [Color figure can be viewed at wileyonlinelibrary.com]

The analysis of OS by cytogenetic change from baseline revealed significant differences across the various groups (log‐rank *p*‐value < 0.01) (Figure 2 and Table S4). Patients who maintained normal cytogenetics (NL‐Cy to NL‐Cy) had the highest 12‐month OS probability at 73% (95% CI: 67–79), and patients with persistent or newly acquired cytogenetic abnormalities (NL/Abnl‐Cy to Gain‐Cy) had the lowest 12‐month OS at 53% (95% CI: 40–70). The multivariable CPH analysis for OS based on cytogenetic changes from baseline revealed significant differences in survival outcomes across the groups (*p* = 0.038) (Table 2). The NL‐Cy to NL‐Cy category served as the reference group in this model. The NL/Abnl‐Cy to Gain‐Cy group had a trend toward worse OS with a Hazard Ratio (HR) of 1.54 (95% CI: 0.99–2.39), though this result was not statistically significant. The Abnl‐Cy to Persistent‐Cy group had the highest HR of 1.61 (95% CI: 1.13–2.31). Conversely, the Abnl‐Cy to Partial‐Cy group and Abnl‐Cy to NL‐Cy group did not have different OS compared to the reference group (HR: 0.76, 95% CI: 0.39–1.49) (HR: 1.25, 95% CI: 0.93–1.68), respectively.

**TABLE 2:** Multivariable Cox regression for overall survival from 60‐day landmark evaluation in acute myeloid leukemia patients stratified by baseline and response cytogenetic state (analytic Scheme 1).

Characteristic	HR	95% CI	*p*
Cytogenetic change from baseline			
NL‐Cy to NL‐Cy	—	—	0.038
NL/Abnl‐Cy to Gain‐Cy	1.54	0.99, 2.39	
Abnl‐Cy to Persistent‐Cy	1.61	1.13, 2.31	
Abnl‐Cy to Partial‐Cy	0.76	0.39, 1.49	
Abnl‐Cy to NL‐Cy	1.25	0.93, 1.68	
Age at first diagnosis (years)	1.04	1.03, 1.05	< 0.001
Gender			
Female	—	—	0.5
Male	0.92	0.73, 1.17	
Coronary artery disease or cerebrovascular disease			
No	—	—	0.2
Yes	1.31	0.91, 1.87	
Chronic heart failure			
No	—	—	0.14
Yes	0.71	0.44, 1.14	
Diabetes			
No	—	—	0.7
Yes	1.06	0.77, 1.46	
Hypertension			
No	—	—	0.8
Yes	1.04	0.81, 1.33	
CKD stage 3/4/5/ESRD			
No	—	—	0.003
Yes	0.49	0.30, 0.81	
Baseline *FLT3‐ITD* (PCR or NGS)			
Negative	—	—	0.2
Positive	1.08	0.75, 1.56	
Not tested	0.78	0.58, 1.05	
Baseline *ASXL1*			
WT	—	—	0.050
Mut	1.28	0.85, 1.92	
Not tested	5.56	1.24, 25.0	
Baseline *BCOR*			
WT	—	—	0.4
Mut	0.73	0.44, 1.21	
Not tested	0.70	0.19, 2.50	
Baseline *CEBPA*			
WT	—	—	0.5
Mut	1.05	0.56, 1.98	
Not tested	2.45	0.53, 11.3	
Baseline *EZH2*			
WT	—	—	0.9
Mut	0.93	0.48, 1.79	
Not tested	0.72	0.20, 2.52	
Baseline *RUNX1*			
WT	—	—	0.037
Mut	0.96	0.63, 1.48	
Not tested	3.27	1.11, 9.63	
Baseline *SF3B1*			
WT	—	—	0.4
Mut	0.65	0.34, 1.25	
Not tested	0.78	0.24, 2.53	
Baseline *SRSF2*			
WT	—	—	0.065
Mut	1.24	0.82, 1.89	
Not tested	0.33	0.10, 1.09	
Baseline *STAG2*			
WT	—	—	0.2
Mut	1.46	0.84, 2.56	
Not tested	0.45	0.10, 2.07	
Baseline *TP53*			
WT	—	—	< 0.001
Mut	2.79	1.86, 4.17	
Not tested	1.08	0.43, 2.75	
Baseline *U2AF1*			
WT	—	—	0.3
Mut	1.33	0.73, 2.46	
Not tested	0.62	0.18, 2.09	
Baseline *ZRSR2*			
WT	—	—	0.4
Mut	1.75	0.76, 4.04	
Not tested	0.81	0.21, 3.11	

Abbreviations: Abnl: abnormal; CI, confidence interval; CKD, chronic kidney disease; Cy, cytogenetic; ESRD, end‐stage renal disease; HR, hazard ratio; Mut, mutation; NGS, next‐generation sequencing; NL, normal; PCR, polymerase chain reaction; WT, wild type.

**FIGURE 3:**
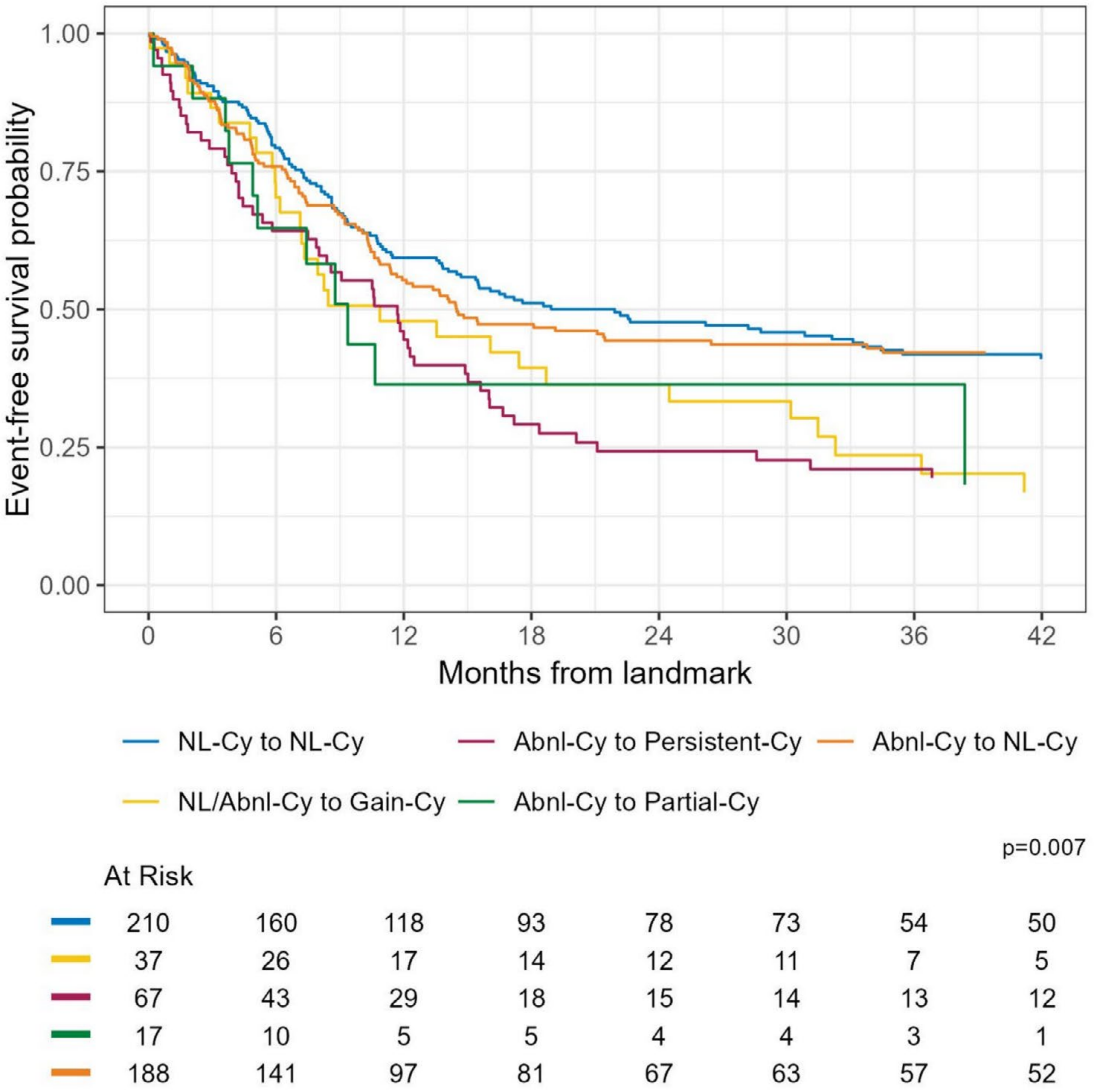
Kaplan–Meier survival curve for event‐free survival in acute myeloid leukemia patients stratified by baseline and response cytogenetic state (analytic Scheme 1). [Color figure can be viewed at wileyonlinelibrary.com]

The EFS outcomes for comparison groups in Scheme 1 are illustrated in Figure 3 and Table S5. Patients who maintained normal cytogenetics (NL‐Cy to NL‐Cy) had the highest 12‐month EFS probability at 59% (95% CI: 53–66) and patients with NL/Abnl‐Cy to Gain‐Cy had a lower 12‐month EFS of 48% (95% CI: 34–67) (log‐rank *p*‐value < 0.01).

For the multivariable CPH analysis for EFS, none of the subgroups were statistically different (Table 3).

**TABLE 3:** Multivariable Cox regression for event‐free survival from 60‐day landmark evaluation in acute myeloid leukemia patients stratified by baseline and response cytogenetic state (analytic Scheme 1).

Characteristic	HR	95% CI	*p*
Cytogenetic change from baseline			
NL‐Cy to NL‐Cy	—	—	0.4
NL/Abnl‐Cy to Gain‐Cy	1.17	0.74, 1.84	
Abnl‐Cy to Persistent‐Cy	1.44	1.00, 2.08	
Abnl‐Cy to Partial‐Cy	1.02	0.53, 1.94	
Abnl‐Cy to NL‐Cy	1.19	0.90, 1.58	
Age at first diagnosis (years)	1.03	1.02, 1.04	< 0.001
Gender			
Female	—	—	0.4
Male	0.91	0.72, 1.15	
Coronary artery disease or cerebrovascular disease			
No	—	—	0.8
Yes	1.05	0.73, 1.51	
Chronic heart failure			
No	—	—	0.3
Yes	0.78	0.48, 1.27	
Diabetes			
No	—	—	0.4
Yes	1.14	0.83, 1.57	
Hypertension			
No	—	—	> 0.9
Yes	1.01	0.78, 1.29	
CKD stage 3/4/5/ESRD			
No	—	—	0.015
Yes	0.57	0.36, 0.92	
Baseline *FLT3‐ITD* (PCR or NGS)			
Negative	—	—	0.049
Positive	1.03	0.72, 1.47	
Not tested	0.69	0.51, 0.94	
Baseline *ASXL1*			
WT	—	—	0.14
Mut	1.20	0.80, 1.82	
Not tested	3.46	0.98, 12.2	
Baseline *BCOR*			
WT	—	—	0.5
Mut	0.74	0.44, 1.26	
Not tested	0.86	0.24, 3.07	
Baseline *CEBPA*			
WT	—	—	0.6
Mut	1.01	0.52, 1.95	
Not tested	1.93	0.53, 6.98	
Baseline *EZH2*			
WT	—	—	> 0.9
Mut	1.13	0.59, 2.16	
Not tested	0.92	0.26, 3.18	
Baseline *RUNX1*			
WT	—	—	0.5
Mut	1.09	0.70, 1.70	
Not tested	1.71	0.65, 4.47	
Baseline *SF3B1*			
WT	—	—	0.2
Mut	0.56	0.28, 1.12	
Not tested	0.61	0.19, 1.98	
Baseline *SRSF2*			
WT	—	—	0.032
Mut	1.26	0.83, 1.91	
Not tested	0.33	0.11, 0.95	
Baseline *STAG2*			
WT	—	—	0.4
Mut	1.27	0.73, 2.21	
Not tested	0.62	0.17, 2.24	
Baseline *TP53*			
WT	—	—	0.001
Mut	2.25	1.47, 3.44	
Not tested	1.30	0.50, 3.37	
Baseline *U2AF1*			
WT	—	—	0.4
Mut	1.33	0.72, 2.45	
Not tested	0.75	0.23, 2.47	
Baseline *ZRSR2*			
WT	—	—	0.5
Mut	1.66	0.71, 3.84	
Not tested	1.57	0.36, 6.80	

Abbreviations: Abnl, abnormal; CI, confidence interval; CKD, chronic kidney disease; Cy, cytogenetic; ESRD, end‐stage renal disease; HR, hazard ratio; Mut, mutation; NGS, next‐generation sequencing; NL, normal; PCR, polymerase chain reaction; WT, wild type.

**FIGURE 4:**
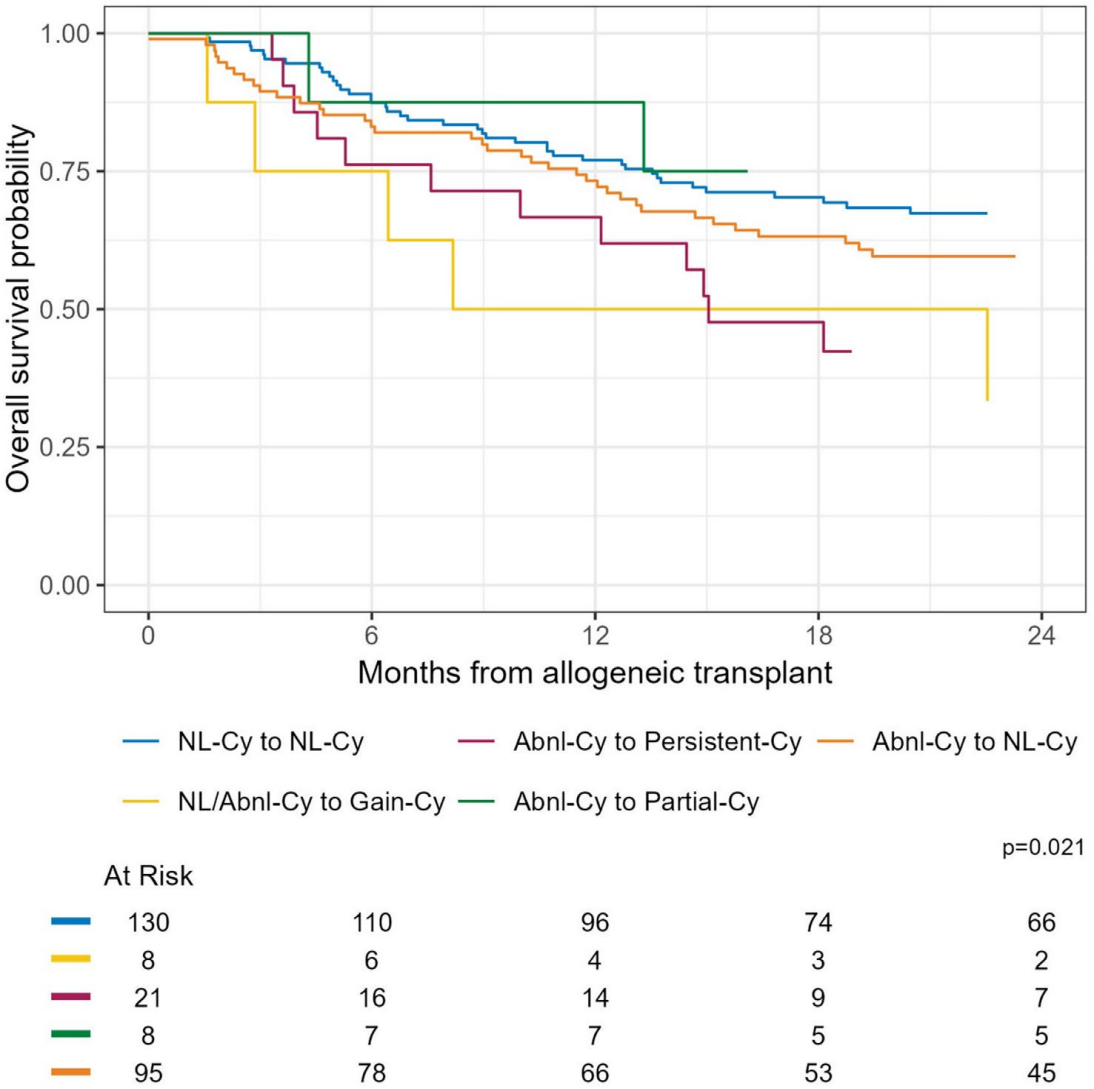
Kaplan–Meier survival curve for overall survival in acute myeloid leukemia patients who received allogeneic transplant, stratified by baseline and response cytogenetic state (analytic Scheme 1). [Color figure can be viewed at wileyonlinelibrary.com]

For OS in allogeneic stem cell transplant recipients, the 6‐month OS for patients with NL‐Cy to NL‐Cy was 87% (95% CI: 82–93) and patients with NL/Abnl‐Cy to Gain‐Cy showed a 6‐month OS of 75% (95% CI: 50–100) (log‐rank *p*‐value = 0.021) (Figure 4 and Table S6).

The multivariable regression CPH analysis for OS following HCT showed significant findings related to cytogenetic changes. The NL/Abnl‐Cy to Gain‐Cy group and the Abnl‐Cy to Persistent‐Cy group had higher mortality than the NL‐Cy to NL‐Cy group (HR: 2.96, 95% CI: 1.23–7.13) (HR: 1.95, 95% CI: 1.05–3.61), respectively. The Abnl‐Cy to NL‐Cy group and Abnl‐Cy to Partial‐Cy group had similar survival to the NL‐Cy to NL‐Cy group (Table S7).

Figure S1 illustrates the comparison groups in this analytic approach. The comparison group counts are summarized in Table S2. On multivariable LR analysis, and using the NL‐Cy to NL‐Cy group as a reference, we found that cytogenetic change from baseline was significantly associated with AML treatment response, such that the NL/Abnl‐Cy to Persistent/Gain‐Cy group had reduced odds of AML response (OR: 0.13, 95% CI: 0.07–0.23) (Table S8), but not the Abnl‐Cy to NL‐Cy group or the Abnl‐Cy to Partial‐Cy group.

The 12‐month OS probability was the lowest in the NL/Abnl‐Cy to Persistent/Gain‐Cy group (log‐rank *p*‐value < 0.001) (Figure S2 and Table S9). In the multivariable CPH analysis for OS (Table S10) using the NL‐Cy to NL‐Cy group as the reference group, cytogenetic change was found to be a significant predictor of survival. The NL/Abnl‐Cy to Persistent/Gain‐Cy group had an HR of 1.59 (95% CI: 1.14–2.21), but there was no difference in OS in the other two groups.

For EFS the 12‐month probability was 59% (95% CI: 53–66) for patients with NL‐Cy to NL‐Cy, compared to 46% (95% CI: 37–56) for those with NL/Abnl‐Cy to Persistent/Gain‐Cy (Figure S3 and Table S11). The multivariable analysis for EFS in Scheme 2 showed that cytogenetic change from baseline was not significantly associated with EFS (Table S12).

Among patients who underwent HCT, cytogenetic response was significantly associated with OS (Figure S4 and Table S13). NL‐Cy to NL‐Cy group had the highest 12‐month OS of 77% (95% CI: 70–85), while those with NL/Abnl‐Cy to Persistent/Gain‐Cy had a 12‐month OS of 62% (95% CI: 47–82) (log‐rank *p*‐value = 0.016). In the multivariable CPH analysis for OS from HCT (Table S14), the NL/Abnl‐Cy to Persistent/Gain‐Cy group had a significantly increased hazard of death (HR: 2.18, 95% CI: 1.2–3.74, *p* = 0.032) compared to the NL‐Cy to NL‐Cy group but not the other groups.

## 5 Discussion

This study offers significant insights into the prognostic value of cytogenetic response in AML, reinforcing its critical role in guiding treatment decisions and shaping long‐term survival outcomes. By evaluating the cytogenetic changes at the 60‐day landmark and using standardized criteria, we demonstrate that cytogenetic normalization, whether complete or partial, is strongly associated with improved OS and not different from patients who have a normal karyotype at baseline and remain normal at response evaluation. These findings emphasize the importance of early cytogenetic assessment as a key determinant of treatment efficacy, highlighting its role in risk stratification and its potential to guide personalized treatment approaches. Using the partial cytogenetic category, we were able to classify patients into a group with improved outcomes compared to previous analytic approaches, which would have categorized this group of patients into persistent cytogenetic abnormalities. Specifically, prior studies have predominantly employed a binary approach, classifying patients according to the presence or absence of cytogenetic abnormalities at the time of CR, without distinguishing between complete clearance and partial reduction of abnormal metaphases [3, 4, 5]. In these analyses, any persistence of cytogenetic abnormalities, regardless of the degree of reduction, was associated with significantly inferior outcomes compared to those who achieved complete cytogenetic remission, with notable differences in relapse‐free survival and OS [3, 4, 5]. By grouping all non‐complete responses together, these studies were unable to determine whether partial cytogenetic response confers an intermediate or possibly favorable prognosis. Our analysis demonstrates that patients achieving Partial‐Cy have survival outcomes comparable to those achieving complete cytogenetic response and superior to those with persistent cytogenetic abnormalities. This distinction is particularly important, as it suggests that even a reduction of cytogenetic abnormalities may signal effective chemosensitivity and translate to improved long‐term outcomes. Partial clearance of abnormal clones may reflect a differential leukemic response to induction therapy, offering an early sign of the disease's underlying biology. Based on our analysis, Partial‐Cy remission, unlike Persistent‐Cy abnormality, should not be interpreted as a sign of increased risk for mortality and refractory disease. However, these findings will need to be replicated before generalization. Furthermore, our study underscores the adverse impact of persistent or acquired cytogenetic abnormalities (i.e., NL/Abnl‐Cy to Persistent/Gain‐Cy), which significantly decreases long‐term survival, even after transplant. Our results align with previous studies that have demonstrated that residual cytogenetic abnormalities are associated with treatment resistance, disease progression, and higher relapse rates. For example, Chen et al. reported a 3‐year OS of 56% for cytogenetic remission versus 15% for persistent abnormalities, and CALGB analyses similarly demonstrated substantially shorter disease‐free and OS for patients with abnormal cytogenetics at CR [3, 4, 5]. Despite the gain of cytogenetic abnormality has been shown to be a frequent finding at times of relapse or refractory disease and an adverse prognostic finding, its significance at first response assessment was not studied before [10].

The LR analysis further confirms the importance of cytogenetic normalization in predicting CR. We found that patients in the NL/Abnl‐Cy to Persistent/Gain‐Cy group had significantly reduced odds of achieving CR, indicating that persistent or new abnormalities serve as reliable markers of treatment failure. This finding emphasizes the need for cytogenetic monitoring after first‐line treatment to identify high‐risk patients who may require alternative or intensified treatments. Importantly, this approach could help clinicians move beyond traditional response criteria, such as morphologic CR, by incorporating cytogenetic response as an additional marker of treatment success and disease control. Future studies are needed to validate the response criteria we used in our study or propose an alternative approach before introducing it to the response criteria guidelines.

EFS analyses in our cohort substantiate the role of cytogenetic response in disease control. Patients with Persistent/Gain‐Cy group had numerically worse EFS than the NL‐Cy to NL‐Cy group. These trends closely follow those reported in landmark cooperative group studies, which defined cytogenetic remission as the state associated with the lowest cumulative incidence of relapse and the best long‐term EFS/DFS [5].

Multivariable CPH confirmed the independent effect of cytogenetic response on both CCR and OS. Cytogenetic change remained prognostic even after adjusting for age, comorbidities, and gene mutations. Specifically, patients in the NL/Abnl‐Cy to Persistent/Gain‐Cy group had a hazard ratio for death of 1.59, consistent with prior literature [3, 4]. Importantly, our results are consistent even among AML patients undergoing HCT. For these individuals, cytogenetic remission, whether partial or complete, predicted excellent one‐year survival, whereas patients with persistent abnormalities had lower survival. Persistently abnormal cytogenetics at first response assessment and prior to transplant were associated with significantly higher post‐transplant mortality, corroborating transplant‐focused studies that identified pre‐HCT cytogenetic status as a critical determinant of post‐transplant outcomes [6, 7, 18]. Taken together, our findings highlight that cytogenetic response is a key dynamic marker of disease biology and treatment efficacy in AML. Early identification of patients with persistent or emergent cytogenetic abnormalities is essential, as these individuals represent a high‐risk group that may benefit from alternative or intensified therapeutic strategies, including investigational agents and targeted molecular therapies. Routine cytogenetic monitoring at early landmarks can provide actionable prognostic information to guide individualized patient management.

While these findings align partially with historical data, certain limitations must be acknowledged. As with prior retrospective analyses, potential confounding factors and treatment heterogeneity may influence outcomes. Despite our study being multicenter, it is confined to one geographical area, which limits its generalizability. Larger observational studies are warranted to further delineate the optimal integration of cytogenetic monitoring and to explore its interaction with emerging molecular MRD techniques. Moreover, further research is needed to validate our findings, especially the response criteria we utilized in our study. Another limitation is that not all patients had NGS done at baseline, which reflects the management of AML in the earlier years of our cohort. Beyond this, conventional cytogenetic analysis is inherently limited by the evaluation of only 20 metaphase cells per sample. As a result, rare abnormal clones present at low frequency might be missed. Moreover, the distinction between partial and persistent cytogenetic response may be affected by sampling variability, as classification is based on a small number of metaphases. This introduces the potential for misclassification between response groups, particularly when the proportion of abnormal cells approaches the response cutoffs. The accuracy of these estimates would improve by increasing the number of examined metaphases (> 20), which decreases sampling error. Additionally, the sensitivity of cytogenetic methods is intrinsically limited by the resolution of chromosome banding; smaller structural alterations or cryptic abnormalities may not be visible by this approach [5, 19]. These technical constraints are not unique to our study but are a recognized challenge in the field [5]. Future studies could address these limitations by incorporating higher‐resolution techniques such as optical genomic mapping, which allows for more comprehensive detection of both rare and submicroscopic chromosomal aberrations [20, 21]. On the other hand, our study has several strengths; it is based on a relatively large sample and covers a period where both conventional chemotherapy and more modern regimens are utilized. Moreover, we used a large number of baseline variables to adjust for confounding, including comorbidities and mutational status of relevant genes, which was not done in prior studies. Lastly, by using standardized criteria, we were able to demonstrate that the partial cytogenetic remission and gain of cytogenetic abnormalities are important events and carry prognostic values, which is a novel finding in our study.

In summary, our study highlights the critical role of cytogenetic response as a prognostic marker in AML. These findings have important clinical implications, emphasizing the need for routine cytogenetic assessment after frontline therapy to guide treatment decisions and optimize long‐term outcomes. The ability to differentiate partial from complete cytogenetic responses provides a more nuanced framework for risk stratification, therapy intensification, and transplant decision‐making. These findings challenge the adequacy of the dichotomous models used in previous foundational studies, which may have underestimated the prognostic benefit for patients achieving a partial cytogenetic response by consolidating them with those experiencing no cytogenetic improvement. By moving beyond the traditional binary categorization and rigorously analyzing partial cytogenetic response as its own entity, our study offers a superior and more clinically informative classification system. Moving forward, observational studies are needed to support these results, validate our response criteria, and investigate the integration of cytogenetic response into risk‐adapted therapeutic strategies. Such efforts could pave the way for more personalized and effective management and prolong survival in AML patients.

## Author Contributions

All authors had full access to all the data and analysis in the study and take responsibility for the integrity of the data and the accuracy of the data analysis. All authors had final responsibility for the decision to submit for publication. Study conception and design: Moaath K. Mustafa Ali and Caroline Astbury. Data Collection: John Hanna, Moath Albliwi, Daniel P. Nurse, Jessica El‐Asmar, Ameed Bawwab, Hasan Abuamsha, Yomna Abu‐Farsakh, Asad Rauf, and Heya Batah. Analysis and Interpretation: All authors. Draft manuscript preparation: All authors. Statistical analysis: Emily C. Zabor. Critical Review of Manuscript: All authors. Administrative and technical support: Moaath K. Mustafa Ali. Supervision: Moaath K. Mustafa Ali.

## Disclosure

Moaath Mustafa Ali affirms that the manuscript is an honest, accurate, and transparent account of the study being reported, that no important aspects of the study have been omitted, and any discrepancies in the study as planned have been explained.

## Conflicts of Interest

The authors declare no conflicts of interest.

## Supporting information


**Data S1.**Supporting Information.

## Data Availability

The data supporting this study's findings are available from Moaath Mustafa Ali upon reasonable request, at moaath_mustafa@yahoo.com.
