# Supplementary material for: Impact of Cytogenetic Response to Therapy on Long‐Term Survival in Acute Myeloid Leukemia
Source: Am J Hematol. 2025 Jul 11;100(9):1577–89. doi: 10.1002/ajh.70000 (PMC12326219; doi:10.1002/ajh.70000)

**Impact of Cytogenetic Response to Therapy on Long-Term Survival in Acute Myeloid Leukemia**

**Supplementary Material**

John Hanna, MD^1^, Emily C. Zabor, DrPH^2^, Moath Albliwi, MD^1^, Jessica El-Asmar, MD, MPH^3^, Daniel P. Nurse, DO^1^, Ameed Bawwab, MD^1^, Hasan Abuamsha, MD^1^, Yomna Abu-Farsakh, MD^3^, Heya Batah, MD^1^, Asad Rauf, MD^1^, Joy Nakitandwe, PhD^4^, David S. Bosler, MD^4^, Akriti G. Jain, MD^3^, John C Molina, MD, Med^3^, Sophia Balderman, MD^3^, Abhay Singh, MD, MPH^3^, Aaron T. Gerds, MD, MS^3^, Sudipto Mukherjee, MD, PhD, MPH^3^, Anjali S. Advani, MD^3^, Hetty E. Carraway, MD, MBA^3^, Caroline Astbury, PhD^4^; Moaath K. Mustafa Ali, MD, MPH^3^

^1^Department of Internal Medicine, Cleveland Clinic, Cleveland, OH; ^2^Department of Quantitative Health Sciences, Cleveland Clinic Taussig Cancer Institute, Cleveland, OH; ^3^Department of Hematology and Medical Oncology, Cleveland Clinic Taussig Cancer Institute, Cleveland, OH; ^4^Department of Pathology and Laboratory Medicine, Cleveland Clinic Diagnostics Institute, Cleveland, OH

Table of Contents

[Methods 2](#_Toc197166448)

[Results 4](#_Toc197166449)

## Methods

**Supplementary Table 1:** Cytogenetic Response Categories for Patients with Acute Myeloid Leukemia Who Received First-Line Therapy and Rationale for Proposed Categories

| Cytogenetic Response | Definition | Reasoning |
| --- | --- | --- |
| Normal Cytogenetic Exam (NL-Cy) | No pathogenic abnormalities were detected at the landmark evaluation. In cases where the exam was suboptimal (i.e., fewer than 20 metaphases analyzed) or based on peripheral blood, it was still classified as NL-Cy for the purpose of this study | Aligns with widely accepted definitions of a normal karyotype. |
| Abnormal Cytogenetic Exam (Abnl-Cy) | Pathogenic abnormalities were identified by the cytogeneticist. Non-pathogenic findings were excluded. This category primarily applied to patients with a normal baseline cytogenetic exam who later developed detectable abnormalities. | Consistent with the commonly used definitions of abnormal karyotypes. |
| Persistent Cytogenetic Abnormalities (Persistent-Cy) | No reduction in abnormal clones and no emergence of normal clones. Defined as ≥50% abnormal metaphases. | The 50% threshold is based on the 2022 ELN response criteria for partial remission (≥50% reduction in blasts from baseline). Using a percentage rather than an absolute count accommodates suboptimal exams with fewer than 20 metaphases. |
| Partial Cytogenetic Remission (Partial-Cy) | A reduction in abnormal clones accompanied by a ≥50% increase in normal clones. Calculated as: normal clones / (normal + abnormal clones) ≥50%. | Mirrors the ELN 2022 criteria for partial response, using percentage-based assessment to adjust for inadequate metaphase counts. |
| Gain of Cytogenetic Abnormality (Gain-Cy) | The emergence of new pathogenic abnormalities not present at baseline. Defined as ≥2 metaphases for gains, rearrangements, or partial losses (e.g., deletions), and ≥3 metaphases for whole chromosome losses (e.g., −5, −7). Typically applied to patients with an abnormal karyotype at baseline. | Captures clonal evolution through the appearance of new pathogenic clones not observed in the baseline cytogenetic exam. |

**Supplementary Figure 1: Comparison of Patient Groups with Acute Myeloid Leukemia Stratified by Baseline and Response Cytogenetic State (Analytic Scheme 2)**

**
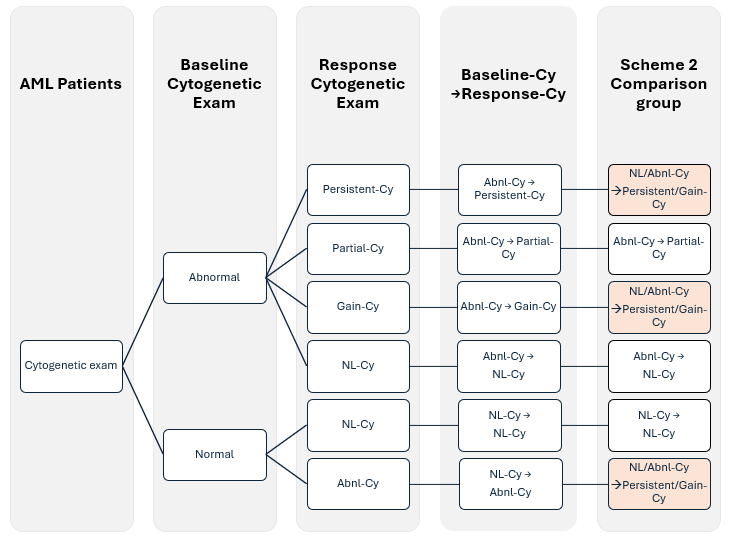
**

Cy: Cytogenetic, NL: Normal, Abnl: Abnormal

## Results

**Supplementary Table 2:** Distribution of Cytogenetic Response at 60-Day Landmark in Acute Myeloid Leukemia Patients Stratified by Baseline and Response Cytogenetic State (Analytic Scheme 1)

| **Characteristic** | **N = 563***^1^* |
| --- | --- |
| **Cytogenetic response** |  |
| *Complete Cytogenetic Remission: Normal Karyotype (no cytogenetic abnormality, > or = 20 metaphase BM) and normal FISH results* | 389 (69%) |
| *Gain of new cytogenetic abnormalities or FISH abnormalities* | 46 (8.2%) |
| *Normal, Inadequate (< 20 metaphase BM)* | 21 (3.7%) |
| *Normal, Suboptimal (> or = 20 metaphase PB)* | 6 (1.1%) |
| *Partial Cytogenetic Remission: Partial improvement in Karyotype and/ or FISH* | 20 (3.6%) |
| *Persistent Cytogenetic and FISH Abnormality* | 81 (14%) |
| **Cytogenetic response** |  |
| *Normal Cytogenetic Exam* | 416 (74%) |
| *Persistent Cytogenetic Abnormalities* | 81 (14%) |
| *Partial Cytogenetic Remission* | 20 (3.6%) |
| *Gain of Cytogenetic Abnormality* | 46 (8.2%) |
| **Cytogenetic change from baseline** |  |
| *NL-Cy to NL-Cy* | 221 (39%) |
| *NL-Cy to Gain-Cy* | 10 (1.8%) |
| *Abnl-Cy to Persistent-Cy* | 81 (14%) |
| *Abnl-Cy to Partial-Cy* | 20 (3.6%) |
| *Abnl-Cy to Gain-Cy* | 36 (6.4%) |
| *Abnl-Cy to NL-Cy* | 195 (35%) |
| **Cytogenetic change from baseline (scheme 1)** |  |
| *NL-Cy to NL-Cy* | 221 (39%) |
| *NL/Abnl-Cy to Gain-Cy* | 46 (8.2%) |
| *Abnl-Cy to Persistent-Cy* | 81 (14%) |
| *Abnl-Cy to Partial-Cy* | 20 (3.6%) |
| *Abnl-Cy to NL-Cy* | 195 (35%) |
| **Cytogenetic change from baseline (scheme 2)** |  |
| *NL-Cy to NL-Cy* | 221 (39%) |
| *NL/Abnl-Cy to Persistent/Gain-Cy* | 127 (23%) |
| *Abnl-Cy to Partial-Cy* | 20 (3.6%) |
| *Abnl-Cy to NL-Cy* | 195 (35%) |

Cy: Cytogenetic, NL: Normal, Abnl: Abnormal, *^1^*n (%)

**Supplementary Table 3:** Multivariable Logistic Regression for Composite Response Rate in First-Line Therapy for Acute Myeloid Leukemia Patients Stratified by Cytogenetic State and Comparison Groups (Analytic Scheme 1)

| **Characteristic** | **OR***^1^* | **95% CI***^1^* | **p-value** |
| --- | --- | --- | --- |
| **Cytogenetic change from baseline** |  |  | <0.001 |
| *NL-Cy to NL-Cy* | — | — |  |
| *NL/Abnl-Cy to Gain-Cy* | 0.09 | 0.04, 0.22 |  |
| *Abnl-Cy to Persistent-Cy* | 0.14 | 0.07, 0.28 |  |
| *Abnl-Cy to Partial-Cy* | 1.21 | 0.38, 4.45 |  |
| *Abnl-Cy to NL-Cy* | 1.50 | 0.86, 2.66 |  |
| **Age at first diagnosis (years)** | 0.98 | 0.96, 1.00 | 0.038 |
| **Gender** |  |  | 0.2 |
| *Female* | — | — |  |
| *Male* | 1.35 | 0.86, 2.13 |  |
| **Coronary artery disease or cerebrovascular disease** |  |  | 0.2 |
| *No* | — | — |  |
| *Yes* | 0.61 | 0.30, 1.24 |  |
| **Chronic heart failure** |  |  | >0.9 |
| *No* | — | — |  |
| *Yes* | 1.06 | 0.40, 2.88 |  |
| **Diabetes** |  |  | 0.10 |
| *No* | — | — |  |
| *Yes* | 1.74 | 0.91, 3.49 |  |
| **Hypertension** |  |  | 0.2 |
| *No* | — | — |  |
| *Yes* | 1.40 | 0.86, 2.32 |  |
| **CKD stage 3/4/5/ESRD** |  |  | >0.9 |
| *No* | — | — |  |
| *Yes* | 1.04 | 0.40, 2.91 |  |
| **Baseline *FLT3-ITD* (PCR or NGS)** |  |  | 0.048 |
| *Negative* | — | — |  |
| *Positive* | 0.76 | 0.40, 1.50 |  |
| *Not tested* | 0.49 | 0.28, 0.87 |  |
| **Baseline *ASXL1*** |  |  | 0.5 |
| *WT* | — | — |  |
| *Mut* | 0.65 | 0.30, 1.41 |  |
| *Not tested* | 0.50 | 0.02, 10.1 |  |
| **Baseline *BCOR*** |  |  | 0.7 |
| *WT* | — | — |  |
| *Mut* | 1.27 | 0.49, 3.55 |  |
| *Not tested* | 2.84 | 0.21, 27.5 |  |
| **Baseline *CEBPA*** |  |  | 0.6 |
| *WT* | — | — |  |
| *Mut* | 1.20 | 0.37, 4.80 |  |
| *Not tested* | 0.20 | 0.00, 3.93 |  |
| **Baseline *EZH2*** |  |  | 0.2 |
| *WT* | — | — |  |
| *Mut* | 2.87 | 0.67, 13.9 |  |
| *Not tested* | 0.53 | 0.02, 6.63 |  |
| **Baseline *RUNX1*** |  |  | 0.2 |
| *WT* | — | — |  |
| *Mut* | 0.49 | 0.24, 1.01 |  |
| *Not tested* | 0.57 | 0.08, 4.54 |  |
| **Baseline *SF3B1*** |  |  | 0.9 |
| *WT* | — | — |  |
| *Mut* | 1.37 | 0.44, 4.56 |  |
| *Not tested* | 1.47 | 0.13, 18.9 |  |
| **Baseline *SRSF2*** |  |  | 0.4 |
| *WT* | — | — |  |
| *Mut* | 0.61 | 0.28, 1.36 |  |
| *Not tested* | 0.28 | 0.02, 3.25 |  |
| **Baseline *STAG2*** |  |  | 0.5 |
| *WT* | — | — |  |
| *Mut* | 0.73 | 0.26, 2.25 |  |
| *Not tested* | 3.82 | 0.19, 258 |  |
| **Baseline *TP53*** |  |  | 0.056 |
| *WT* | — | — |  |
| *Mut* | 0.49 | 0.22, 1.11 |  |
| *Not tested* | 2.84 | 0.39, 28.9 |  |
| **Baseline *U2AF1*** |  |  | 0.9 |
| *WT* | — | — |  |
| *Mut* | 0.79 | 0.25, 2.94 |  |
| *Not tested* | 0.54 | 0.04, 6.92 |  |
| **Baseline *ZRSR2*** |  |  | 0.6 |
| *WT* | — | — |  |
| *Mut* | 0.87 | 0.16, 7.20 |  |
| *Not tested* | 3.86 | 0.21, 106 |  |

Cy: Cytogenetic, NL: Normal, Abnl: Abnormal, CKD: Chronic Kidney Disease, ESRD: End-Stage Renal Disease, PCR: Polymerase Chain Reaction, NGS: Next-Generation Sequencing, WT: Wild Type, Mut: Mutation, *^1^*OR = Odds Ratio, CI = Confidence Interval

**Supplementary Table 4:** Overall Survival (OS) Stratified by Cytogenetic Response Group from 60-Day Landmark Evaluation in Acute Myeloid Leukemia Patients (Analytic Scheme 1)

| **Characteristic** | **12-month OS** | **24-month OS** | **Median OS** |
| --- | --- | --- | --- |
| **Cytogenetic change from baseline** |  |  |  |
| *NL-Cy to NL-Cy* | 73% (67%, 79%) | 57% (51%, 65%) | 37 (27, 91) |
| *NL/Abnl-Cy to Gain-Cy* | 53% (40%, 70%) | 38% (26%, 56%) | 14 (8.6, 30) |
| *Abnl-Cy to Persistent-Cy* | 58% (48%, 70%) | 29% (20%, 42%) | 13 (12, 18) |
| *Abnl-Cy to Partial-Cy* | 78% (60%, 100%) | 53% (34%, 83%) | 25 (14, —) |
| *Abnl-Cy to NL-Cy* | 70% (64%, 77%) | 53% (46%, 61%) | 27 (19, 101) |

Cy: Cytogenetic, NL: Normal, Abnl: Abnormal

**Supplementary Table 5**: Event-Free Survival (EFS) Stratified by Cytogenetic Response Group from 60-Day Landmark Evaluation in Acute Myeloid Leukemia Patients (Analytic Scheme 1)

| **Characteristic** | **12-month EFS** | **24-month EFS** | **Median EFS** |
| --- | --- | --- | --- |
| **Cytogenetic change from baseline** |  |  |  |
| *NL-Cy to NL-Cy* | 59% (53%, 66%) | 48% (41%, 55%) | 22 (14, 35) |
| *NL/Abnl-Cy to Gain-Cy* | 48% (34%, 67%) | 36% (24%, 56%) | 11 (7.2, 31) |
| *Abnl-Cy to Persistent-Cy* | 44% (34%, 58%) | 24% (16%, 37%) | 12 (8.0, 16) |
| *Abnl-Cy to Partial-Cy* | 36% (19%, 71%) | 36% (19%, 71%) | 9.4 (5.1, —) |
| *Abnl-Cy to NL-Cy* | 55% (49%, 63%) | 44% (38%, 52%) | 14 (11, 123) |

Cy: Cytogenetic, NL: Normal, Abnl: Abnormal

**Supplementary Table 6**: Overall Survival (OS) in Allogeneic Stem Cell Transplant Recipients Stratified by Cytogenetic Response in Acute Myeloid Leukemia Patients (Analytic Scheme 1)

| **Characteristic** | **6-month OS** | **12-month OS** | **Median OS** |
| --- | --- | --- | --- |
| **Cytogenetic change from baseline** |  |  |  |
| *NL-Cy to NL-Cy* | 87% (82%, 93%) | 77% (70%, 85%) | 87 (41, —) |
| *NL/Abnl-Cy to Gain-Cy* | 75% (50%, 100%) | 50% (25%, 100%) | 15 (6.4, —) |
| *Abnl-Cy to Persistent-Cy* | 76% (60%, 97%) | 67% (49%, 90%) | 15 (10, —) |
| *Abnl-Cy to Partial-Cy* | 88% (67%, 100%) | 88% (67%, 100%) | — (—, —) |
| *Abnl-Cy to NL-Cy* | 83% (76%, 91%) | 73% (65%, 83%) | — (25, —) |

Cy: Cytogenetic, NL: Normal, Abnl: Abnormal

**Supplementary Table 7:** Multivariable Cox Regression for Overall Survival in Allogeneic Stem Cell Transplant Recipients with Acute Myeloid Leukemia Stratified by Cytogenetic Response (Analytic Scheme 1)

| **Characteristic** | **HR***^1^* | **95% CI***^1^* | **p-value** |
| --- | --- | --- | --- |
| **Cytogenetic change from baseline** |  |  | 0.051 |
| *NL-Cy to NL-Cy* | — | — |  |
| *NL/Abnl-Cy to Gain-Cy* | 2.96 | 1.23, 7.13 |  |
| *Abnl-Cy to Persistent-Cy* | 1.95 | 1.05, 3.61 |  |
| *Abnl-Cy to Partial-Cy* | 0.55 | 0.13, 2.31 |  |
| *Abnl-Cy to NL-Cy* | 1.27 | 0.82, 1.97 |  |
| **Age at first dx (yrs)** | 1.01 | 1.00, 1.03 | 0.078 |
| **Gender** |  |  | 0.8 |
| *Female* | — | — |  |
| *Male* | 1.04 | 0.71, 1.53 |  |
| **Coronary artery disease or cerebrovascular disease** |  |  | 0.9 |
| *No* | — | — |  |
| *Yes* | 1.06 | 0.54, 2.09 |  |
| **Chronic heart failure** |  |  | >0.9 |
| *No* | — | — |  |
| *Yes* | 1.02 | 0.47, 2.23 |  |
| **Diabetes** |  |  | 0.6 |
| *No* | — | — |  |
| *Yes* | 1.15 | 0.67, 1.99 |  |
| **Hypertension** |  |  | 0.8 |
| *No* | — | — |  |
| *Yes* | 0.93 | 0.60, 1.46 |  |
| **CKD stage 3/4/5/ESRD** |  |  | 0.3 |
| *No* | — | — |  |
| *Yes* | 1.55 | 0.73, 3.29 |  |
| **Baseline *FLT3-ITD* (PCR or NGS)** |  |  | 0.7 |
| *Negative* | — | — |  |
| *Positive* | 0.79 | 0.45, 1.39 |  |
| *Not tested* | 0.92 | 0.53, 1.59 |  |

Cy: Cytogenetic, NL: Normal, Abnl: Abnormal, CKD: Chronic Kidney Disease, ESRD: End-Stage Renal Disease, PCR: Polymerase Chain Reaction, NGS: Next-Generation Sequencing, *^1^*HR = Hazard Ratio, CI = Confidence Interval

**Supplementary Table 8:** Multivariable Logistic Regression for Composite Response Rate in First-Line Therapy for Acute Myeloid Leukemia Patients Stratified by Cytogenetic State and Comparison Groups (Analytic Scheme 2)

| **Characteristic** | **OR***^1^* | **95% CI***^1^* | **p-value** |
| --- | --- | --- | --- |
| **Cytogenetic change from baseline** |  |  | <0.001 |
| *NL-Cy to NL-Cy* | — | — |  |
| *NL/Abnl-Cy to Persistent/Gain-Cy* | 0.13 | 0.07, 0.23 |  |
| *Abnl-Cy to Partial-Cy* | 1.22 | 0.38, 4.49 |  |
| *Abnl-Cy to NL-Cy* | 1.49 | 0.85, 2.65 |  |
| **Age at first diagnosis (years)** | 0.98 | 0.96, 1.00 | 0.033 |
| **Gender** |  |  | 0.2 |
| *Female* | — | — |  |
| *Male* | 1.36 | 0.86, 2.14 |  |
| **Coronary artery disease or cerebrovascular disease** |  |  | 0.2 |
| *No* | — | — |  |
| *Yes* | 0.62 | 0.31, 1.26 |  |
| **Chronic heart failure** |  |  | >0.9 |
| *No* | — | — |  |
| *Yes* | 1.03 | 0.39, 2.77 |  |
| **Diabetes** |  |  | 0.10 |
| *No* | — | — |  |
| *Yes* | 1.72 | 0.90, 3.43 |  |
| **Hypertension** |  |  | 0.2 |
| *No* | — | — |  |
| *Yes* | 1.40 | 0.85, 2.31 |  |
| **CKD stage 3/4/5/ESRD** |  |  | >0.9 |
| *No* | — | — |  |
| *Yes* | 1.05 | 0.40, 2.92 |  |
| **Baseline *FLT3-ITD* (PCR or NGS)** |  |  | 0.048 |
| *Negative* | — | — |  |
| *Positive* | 0.75 | 0.39, 1.48 |  |
| *Not tested* | 0.49 | 0.28, 0.87 |  |
| **Baseline *ASXL1*** |  |  | 0.5 |
| *WT* | — | — |  |
| *Mut* | 0.65 | 0.30, 1.42 |  |
| *Not tested* | 0.45 | 0.02, 8.98 |  |
| **Baseline *BCOR*** |  |  | 0.7 |
| *WT* | — | — |  |
| *Mut* | 1.28 | 0.49, 3.56 |  |
| *Not tested* | 2.55 | 0.19, 24.1 |  |
| **Baseline *CEBPA*** |  |  | 0.5 |
| *WT* | — | — |  |
| *Mut* | 1.19 | 0.37, 4.69 |  |
| *Not tested* | 0.19 | 0.00, 3.70 |  |
| **Baseline *EZH2*** |  |  | 0.2 |
| *WT* | — | — |  |
| *Mut* | 2.81 | 0.66, 13.4 |  |
| *Not tested* | 0.50 | 0.02, 6.16 |  |
| **Baseline *RUNX1*** |  |  | 0.15 |
| *WT* | — | — |  |
| *Mut* | 0.48 | 0.23, 1.01 |  |
| *Not tested* | 0.58 | 0.08, 4.62 |  |
| **Baseline *SF3B1*** |  |  | 0.9 |
| *WT* | — | — |  |
| *Mut* | 1.34 | 0.43, 4.39 |  |
| *Not tested* | 1.45 | 0.13, 18.4 |  |
| **Baseline *SRSF2*** |  |  | 0.4 |
| *WT* | — | — |  |
| *Mut* | 0.61 | 0.28, 1.35 |  |
| *Not tested* | 0.29 | 0.02, 3.40 |  |
| **Baseline *STAG2*** |  |  | 0.5 |
| *WT* | — | — |  |
| *Mut* | 0.72 | 0.25, 2.22 |  |
| *Not tested* | 4.02 | 0.20, 276 |  |
| **Baseline *TP53*** |  |  | 0.050 |
| *WT* | — | — |  |
| *Mut* | 0.48 | 0.21, 1.07 |  |
| *Not tested* | 2.74 | 0.37, 27.9 |  |
| **Baseline *U2AF1*** |  |  | >0.9 |
| *WT* | — | — |  |
| *Mut* | 0.80 | 0.25, 2.96 |  |
| *Not tested* | 0.67 | 0.06, 7.99 |  |
| **Baseline *ZRSR2*** |  |  | 0.6 |
| *WT* | — | — |  |
| *Mut* | 0.89 | 0.16, 7.53 |  |
| *Not tested* | 4.09 | 0.22, 111 |  |

Cy: Cytogenetic, NL: Normal, Abnl: Abnormal CKD: Chronic Kidney Disease, ESRD: End-Stage Renal Disease, PCR: Polymerase Chain Reaction, NGS: Next-Generation Sequencing, WT: Wild Type, Mut: Mutation, *^1^*OR: Odds Ratio, CI: Confidence Interval

**Supplementary Table 9:** Overall Survival (OS) by Cytogenetic Response Group from 60-Day Landmark Evaluation in Acute Myeloid Leukemia Patients (Analytic Scheme 2)

| **Characteristic** | **12-month OS** | **24-month OS** | **Median OS** |
| --- | --- | --- | --- |
| **Cytogenetic change from baseline** |  |  |  |
| *NL-Cy to NL-Cy* | 73% (67%, 79%) | 57% (51%, 65%) | 37 (27, 91) |
| *NL/Abnl-Cy to Persistent/Gain-Cy* | 56% (48%, 66%) | 32% (25%, 42%) | 13 (12, 17) |
| *Abnl-Cy to Partial-Cy* | 78% (60%, 100%) | 53% (34%, 83%) | 25 (14, —) |
| *Abnl-Cy to NL-Cy* | 70% (64%, 77%) | 53% (46%, 61%) | 27 (19, 101) |

Cy: Cytogenetic, NL: Normal, Abnl: Abnormal

**Supplementary Table 10:** Multivariable Cox Regression for Overall Survival from 60-Day Landmark Evaluation in Acute Myeloid Leukemia Patients Stratified by Baseline and Response Cytogenetic State (Analytic Scheme 2)

| **Characteristic** | **HR***^1^* | **95% CI***^1^* | **p-value** |
| --- | --- | --- | --- |
| **Cytogenetic change from baseline** |  |  | 0.018 |
| *NL-Cy to NL-Cy* | — | — |  |
| *NL/Abnl-Cy to Persistent/Gain-Cy* | 1.59 | 1.14, 2.21 |  |
| *Abnl-Cy to Partial-Cy* | 0.76 | 0.39, 1.49 |  |
| *Abnl-Cy to NL-Cy* | 1.25 | 0.93, 1.68 |  |
| **Age at first diagnosis (years)** | 1.04 | 1.03, 1.05 | <0.001 |
| **Gender** |  |  | 0.5 |
| *Female* | — | — |  |
| *Male* | 0.92 | 0.73, 1.17 |  |
| **Coronary artery disease or cerebrovascular disease** |  |  | 0.2 |
| *No* | — | — |  |
| *Yes* | 1.31 | 0.91, 1.87 |  |
| **Chronic heart failure** |  |  | 0.14 |
| *No* | — | — |  |
| *Yes* | 0.71 | 0.44, 1.13 |  |
| **Diabetes** |  |  | 0.7 |
| *No* | — | — |  |
| *Yes* | 1.06 | 0.77, 1.46 |  |
| **Hypertension** |  |  | 0.8 |
| *No* | — | — |  |
| *Yes* | 1.04 | 0.81, 1.33 |  |
| **CKD stage 3/4/5/ESRD** |  |  | 0.003 |
| *No* | — | — |  |
| *Yes* | 0.49 | 0.30, 0.81 |  |
| **Baseline *FLT3-ITD* (PCR or NGS)** |  |  | 0.2 |
| *Negative* | — | — |  |
| *Positive* | 1.08 | 0.75, 1.55 |  |
| *Not tested* | 0.78 | 0.58, 1.05 |  |
| **Baseline *ASXL1*** |  |  | 0.050 |
| *WT* | — | — |  |
| *Mut* | 1.28 | 0.85, 1.92 |  |
| *Not tested* | 5.54 | 1.23, 24.9 |  |
| **Baseline *BCOR*** |  |  | 0.4 |
| *WT* | — | — |  |
| *Mut* | 0.73 | 0.44, 1.21 |  |
| *Not tested* | 0.68 | 0.19, 2.40 |  |
| **Baseline *CEBPA*** |  |  | 0.5 |
| *WT* | — | — |  |
| *Mut* | 1.05 | 0.56, 1.98 |  |
| *Not tested* | 2.43 | 0.53, 11.2 |  |
| **Baseline *EZH2*** |  |  | 0.9 |
| *WT* | — | — |  |
| *Mut* | 0.93 | 0.48, 1.79 |  |
| *Not tested* | 0.71 | 0.20, 2.50 |  |
| **Baseline *RUNX1*** |  |  | 0.036 |
| *WT* | — | — |  |
| *Mut* | 0.96 | 0.63, 1.48 |  |
| *Not tested* | 3.29 | 1.12, 9.69 |  |
| **Baseline *SF3B1*** |  |  | 0.4 |
| *WT* | — | — |  |
| *Mut* | 0.65 | 0.34, 1.25 |  |
| *Not tested* | 0.79 | 0.25, 2.53 |  |
| **Baseline *SRSF2*** |  |  | 0.066 |
| *WT* | — | — |  |
| *Mut* | 1.24 | 0.82, 1.89 |  |
| *Not tested* | 0.34 | 0.10, 1.09 |  |
| **Baseline *STAG2*** |  |  | 0.2 |
| *WT* | — | — |  |
| *Mut* | 1.47 | 0.84, 2.57 |  |
| *Not tested* | 0.45 | 0.10, 2.07 |  |
| **Baseline *TP53*** |  |  | <0.001 |
| *WT* | — | — |  |
| *Mut* | 2.79 | 1.86, 4.17 |  |
| *Not tested* | 1.08 | 0.43, 2.73 |  |
| **Baseline *U2AF1*** |  |  | 0.3 |
| *WT* | — | — |  |
| *Mut* | 1.34 | 0.73, 2.46 |  |
| *Not tested* | 0.63 | 0.19, 2.09 |  |
| **Baseline *ZRSR2*** |  |  | 0.4 |
| *WT* | — | — |  |
| *Mut* | 1.76 | 0.76, 4.05 |  |
| *Not tested* | 0.81 | 0.21, 3.11 |  |

Cy: Cytogenetic, NL: Normal, Abnl: Abnormal, CKD: Chronic Kidney Disease, ESRD: End-Stage Renal Disease, PCR: Polymerase Chain Reaction, NGS: Next-Generation Sequencing, WT: Wild Type, Mut: Mutation, *^1^*HR = Hazard Ratio, CI = Confidence Interval

**Supplementary Table 11**: Event-Free Survival (EFS) Stratified by Cytogenetic Response Group from 60-Day Landmark Evaluation in Acute Myeloid Leukemia (Analytic Scheme 2)

| **Characteristic** | **12-month EFS** | **24-month EFS** | **Median EFS** |
| --- | --- | --- | --- |
| **Cytogenetic change from baseline** |  |  |  |
| *NL-Cy to NL-Cy* | 59% (53%, 66%) | 48% (41%, 55%) | 22 (14, 35) |
| *NL/Abnl-Cy to Persistent/Gain-Cy* | 46% (37%, 56%) | 29% (21%, 39%) | 11 (8.0, 16) |
| *Abnl-Cy to Partial-Cy* | 36% (19%, 71%) | 36% (19%, 71%) | 9.4 (5.1, —) |
| *Abnl-Cy to NL-Cy* | 55% (49%, 63%) | 44% (38%, 52%) | 14 (11, 123) |

Cy: Cytogenetic, NL: Normal, Abnl: Abnormal

**Supplementary Table 12**: Multivariable Cox Regression for Event-Free Survival from 60-Day Landmark Evaluation in Acute Myeloid Leukemia Patients Stratified by Baseline and Response Cytogenetic State (Analytic Scheme 2)

| **Characteristic** | **HR***^1^* | **95% CI***^1^* | **p-value** |
| --- | --- | --- | --- |
| **Cytogenetic change from baseline** |  |  | 0.3 |
| *NL-Cy to NL-Cy* | — | — |  |
| *NL/Abnl-Cy to Persistent/Gain-Cy* | 1.34 | 0.96, 1.88 |  |
| *Abnl-Cy to Partial-Cy* | 1.02 | 0.53, 1.94 |  |
| *Abnl-Cy to NL-Cy* | 1.19 | 0.89, 1.57 |  |
| **Age at first diagnosis (years)** | 1.03 | 1.02, 1.04 | <0.001 |
| **Gender** |  |  | 0.4 |
| *Female* | — | — |  |
| *Male* | 0.91 | 0.72, 1.15 |  |
| **Coronary artery disease or cerebrovascular disease** |  |  | 0.8 |
| *No* | — | — |  |
| *Yes* | 1.06 | 0.73, 1.52 |  |
| **Chronic heart failure** |  |  | 0.3 |
| *No* | — | — |  |
| *Yes* | 0.76 | 0.47, 1.24 |  |
| **Diabetes** |  |  | 0.4 |
| *No* | — | — |  |
| *Yes* | 1.14 | 0.83, 1.57 |  |
| **Hypertension** |  |  | >0.9 |
| *No* | — | — |  |
| *Yes* | 1.01 | 0.78, 1.29 |  |
| **CKD stage 3/4/5/ESRD** |  |  | 0.013 |
| *No* | — | — |  |
| *Yes* | 0.57 | 0.35, 0.91 |  |
| **Baseline *FLT3-ITD* (PCR or NGS)** |  |  | 0.055 |
| *Negative* | — | — |  |
| *Positive* | 1.02 | 0.72, 1.47 |  |
| *Not tested* | 0.70 | 0.52, 0.94 |  |
| **Baseline *ASXL1*** |  |  | 0.14 |
| *WT* | — | — |  |
| *Mut* | 1.22 | 0.81, 1.83 |  |
| *Not tested* | 3.41 | 0.96, 12.1 |  |
| **Baseline *BCOR*** |  |  | 0.5 |
| *WT* | — | — |  |
| *Mut* | 0.73 | 0.43, 1.25 |  |
| *Not tested* | 0.79 | 0.23, 2.73 |  |
| **Baseline *CEBPA*** |  |  | 0.6 |
| *WT* | — | — |  |
| *Mut* | 1.00 | 0.52, 1.93 |  |
| *Not tested* | 1.85 | 0.52, 6.59 |  |
| **Baseline *EZH2*** |  |  | 0.9 |
| *WT* | — | — |  |
| *Mut* | 1.15 | 0.60, 2.20 |  |
| *Not tested* | 0.91 | 0.26, 3.15 |  |
| **Baseline *RUNX1*** |  |  | 0.5 |
| *WT* | — | — |  |
| *Mut* | 1.08 | 0.69, 1.69 |  |
| *Not tested* | 1.72 | 0.66, 4.51 |  |
| **Baseline *SF3B1*** |  |  | 0.2 |
| *WT* | — | — |  |
| *Mut* | 0.56 | 0.28, 1.12 |  |
| *Not tested* | 0.64 | 0.20, 2.03 |  |
| **Baseline *SRSF2*** |  |  | 0.031 |
| *WT* | — | — |  |
| *Mut* | 1.27 | 0.84, 1.92 |  |
| *Not tested* | 0.33 | 0.11, 0.96 |  |
| **Baseline *STAG2*** |  |  | 0.4 |
| *WT* | — | — |  |
| *Mut* | 1.29 | 0.74, 2.24 |  |
| *Not tested* | 0.64 | 0.18, 2.27 |  |
| **Baseline *TP53*** |  |  | 0.001 |
| *WT* | — | — |  |
| *Mut* | 2.25 | 1.47, 3.45 |  |
| *Not tested* | 1.27 | 0.49, 3.27 |  |
| **Baseline *U2AF1*** |  |  | 0.5 |
| *WT* | — | — |  |
| *Mut* | 1.34 | 0.73, 2.46 |  |
| *Not tested* | 0.83 | 0.26, 2.67 |  |
| **Baseline *ZRSR2*** |  |  | 0.5 |
| *WT* | — | — |  |
| *Mut* | 1.66 | 0.71, 3.85 |  |
| *Not tested* | 1.55 | 0.36, 6.66 |  |

Cy: Cytogenetic, NL: Normal, Abnl: Abnormal

Cy: Cytogenetic, NL: Normal, Abnl: Abnormal, CKD: Chronic Kidney Disease, ESRD: End-Stage Renal Disease, PCR: Polymerase Chain Reaction, NGS: Next-Generation Sequencing, WT: Wild Type, Mut: Mutation, *^1^*HR = Hazard Ratio, CI = Confidence Interval

**Supplementary Table 13**: Overall Survival (OS) in Allogeneic Stem Cell Transplant Recipients Stratified by Cytogenetic Response in Acute Myeloid Leukemia (Analytic Scheme 2)

| **Characteristic** | **6-month OS** | **12-month OS** | **Median OS** |
| --- | --- | --- | --- |
| **Cytogenetic change from baseline** |  |  |  |
| *NL-Cy to NL-Cy* | 87% (82%, 93%) | 77% (70%, 85%) | 87 (41, —) |
| *NL/Abnl-Cy to Persistent/Gain-Cy* | 76% (62%, 93%) | 62% (47%, 82%) | 15 (10, —) |
| *Abnl-Cy to Partial-Cy* | 88% (67%, 100%) | 88% (67%, 100%) | — (—, —) |
| *Abnl-Cy to NL-Cy* | 83% (76%, 91%) | 73% (65%, 83%) | — (25, —) |

Cy: Cytogenetic, NL: Normal, Abnl: Abnormal

**Supplementary Table 14:** Multivariable Cox Regression for Overall Survival in Allogeneic Stem Cell Transplant Recipients Stratified by Cytogenetic Response (Analytic Scheme 2)

| **Characteristic** | **HR***^1^* | **95% CI***^1^* | **p-value** |
| --- | --- | --- | --- |
| **Cytogenetic change from baseline** |  |  | 0.032 |
| *NL-Cy to NL-Cy* | — | — |  |
| *NL/Abnl-Cy to Persistent/Gain-Cy* | 2.18 | 1.27, 3.74 |  |
| *Abnl-Cy to Partial-Cy* | 0.55 | 0.13, 2.30 |  |
| *Abnl-Cy to NL-Cy* | 1.27 | 0.82, 1.97 |  |
| **Age at first diagnosis (years)** | 1.01 | 1.00, 1.03 | 0.078 |
| **Gender** |  |  | >0.9 |
| *Female* | — | — |  |
| *Male* | 1.02 | 0.70, 1.51 |  |
| **Coronary artery disease or cerebrovascular disease** |  |  | >0.9 |
| *No* | — | — |  |
| *Yes* | 1.02 | 0.52, 1.99 |  |
| **Chronic heart failure** |  |  | 0.9 |
| *No* | — | — |  |
| *Yes* | 1.06 | 0.49, 2.29 |  |
| **Diabetes** |  |  | 0.6 |
| *No* | — | — |  |
| *Yes* | 1.16 | 0.68, 2.00 |  |
| **Hypertension** |  |  | 0.8 |
| *No* | — | — |  |
| *Yes* | 0.94 | 0.60, 1.47 |  |
| **CKD stage 3/4/5/ESRD** |  |  | 0.3 |
| *No* | — | — |  |
| *Yes* | 1.56 | 0.73, 3.31 |  |
| **Baseline *FLT3-ITD* (PCR or NGS)** |  |  | 0.7 |
| *Negative* | — | — |  |
| *Positive* | 0.80 | 0.45, 1.39 |  |
| *Not tested* | 0.90 | 0.53, 1.55 |  |

Cy: Cytogenetic, NL: Normal, Abnl: Abnormal, CKD: Chronic Kidney Disease, ESRD: End-Stage Renal Disease, PCR: Polymerase Chain Reaction, NGS: Next-Generation Sequencing, *^1^*HR = Hazard Ratio, CI = Confidence Interval

**Supplementary Figure 2: Kaplan-Meier Survival Curve for Overall Survival in Acute Myeloid Leukemia Patients Stratified by Baseline and Response Cytogenetic State (Analytic Scheme 2)**

**
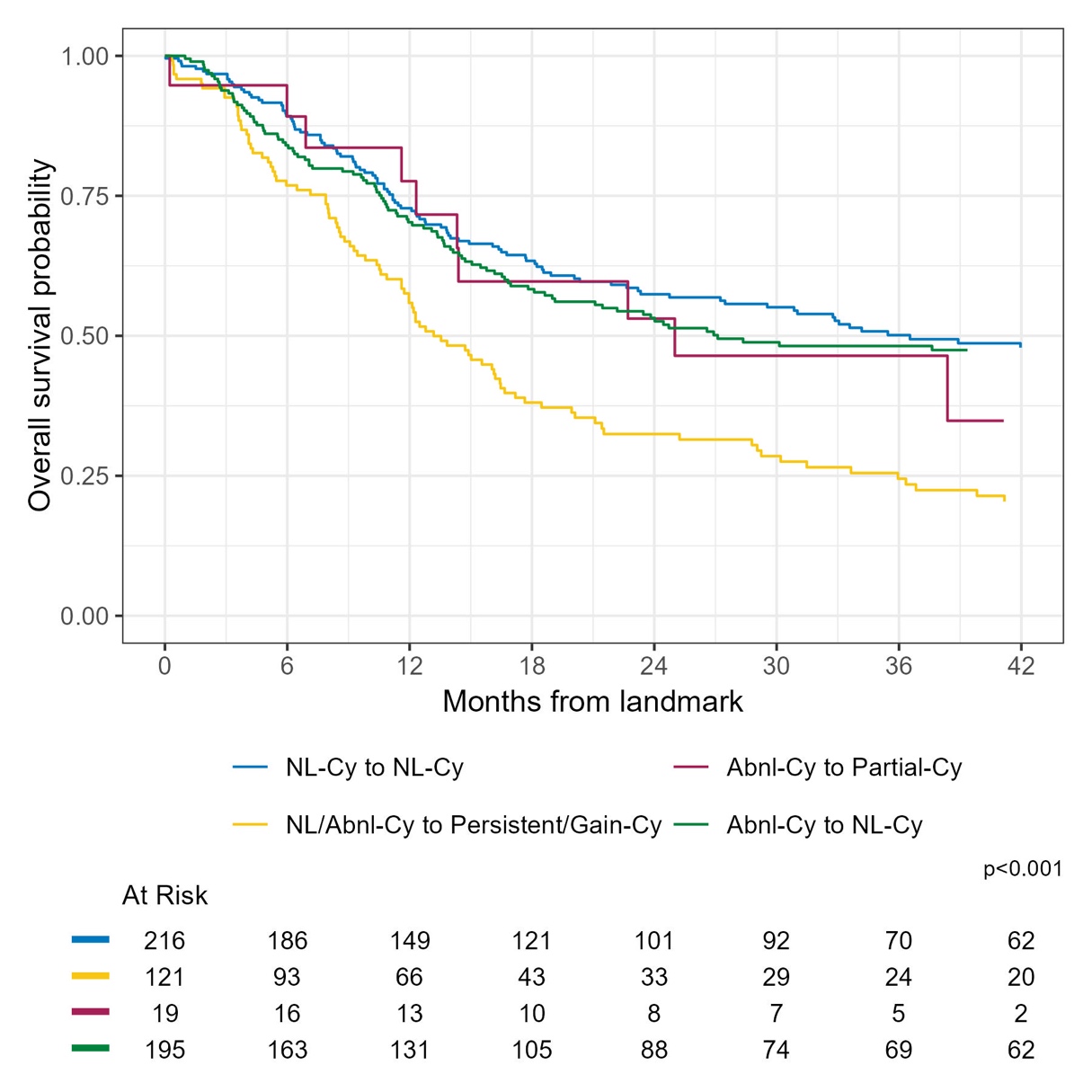
**

**Supplementary Figure 3: Kaplan-Meier Survival Curve for Event-Free Survival in Acute Myeloid Leukemia Patients Stratified by Baseline and Response Cytogenetic State (Analytic Scheme 2)**

**
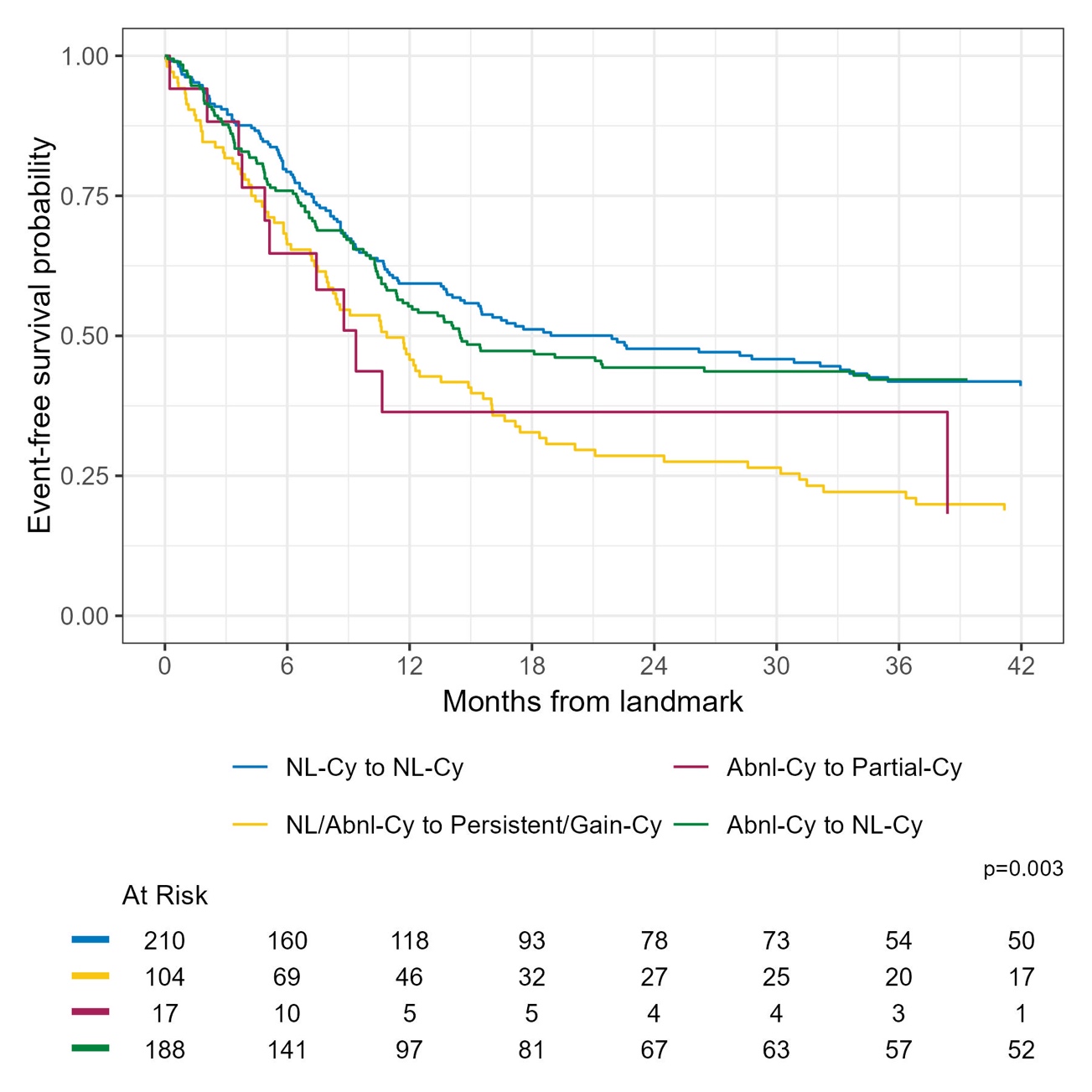
**

**Supplementary Figure 4: Kaplan-Meier Survival Curve for Overall Survival in Acute Myeloid Leukemia Patients Who Received Allogeneic Transplant, Stratified by Baseline and Response Cytogenetic State (Analytic Scheme 2)**


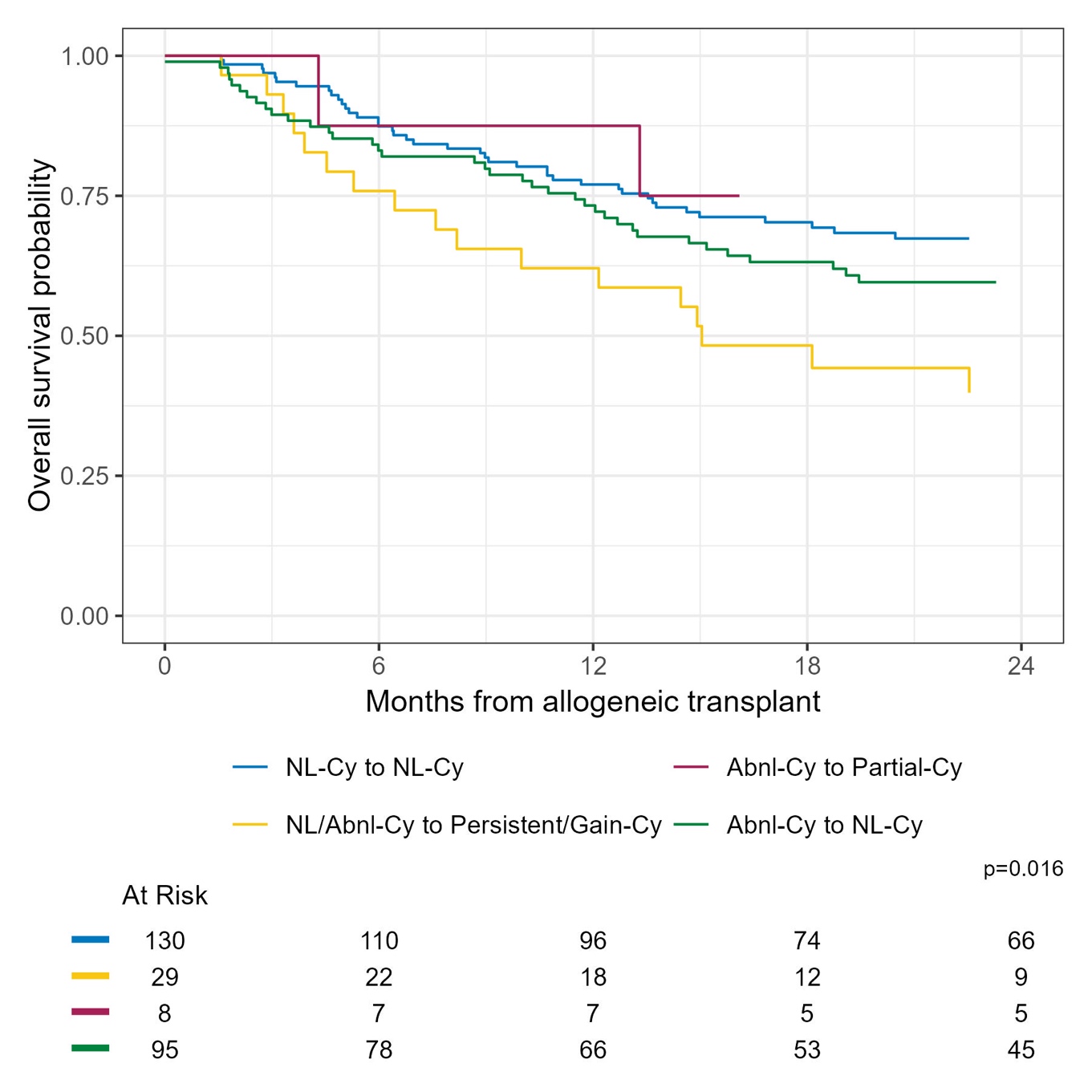

Supplement: Supplementary file 1 — Data S1.Supporting Information. [file AJH-100-1577-s001.docx]
